# Hepatocyte-specific TMEM16A deficiency alleviates hepatic ischemia/reperfusion injury via suppressing GPX4-mediated ferroptosis

**DOI:** 10.1038/s41419-022-05518-w

**Published:** 2022-12-26

**Authors:** Jiawei Guo, Zihao Song, Jie Yu, Chengyi Li, Chenchen Jin, Wei Duan, Xiu Liu, Yingying Liu, Shuai Huang, Yonghua Tuo, Fei Pei, Zhengyang Jian, Pengyu Zhou, Shaoyi Zheng, Zhaowei Zou, Feng Zhang, Quan Gong, Sijia Liang

**Affiliations:** 1 Department of Pharmacology, School of Medicine, Yangtze University, Jingzhou, China; 2 Department of Immunology, School of Medicine, Yangtze University, Jingzhou, China; 3 Department of General Surgery, Zhujiang Hospital, Southern Medical University, Guangzhou, China; 4 Center for Neuro-Metabolism and Regeneration Research, The Bioland Laboratory, Guangzhou, China; 5 Department of Oncology, Jingzhou Hospital Affiliated to Yangtze University, Jingzhou, China; 6 Department of Cardiovascular Surgery, Nanfang Hospital, Southern Medical University, Guangzhou, China; 7 Guangzhou Women and Children’s Medical Center, Guangdong Provincial Clinical Research Center for Child Health, Guangzhou, China; 8 Department of Orthopaedic Surgery, The Second Affiliated Hospital of Guangzhou Medical University, Guangzhou, China; 9 Department of Neurosurgery, The Second Affiliated Hospital of Guangzhou Medical University, Guangzhou, China; 10 Department of Critical Care Medicine, The First Affiliated Hospital, Sun Yat-Sen University, Guangzhou, China; 11 Guangdong Clinical Research Center for Critical Care Medicine, Guangzhou, China; 12 Center For Drug Inspection of Guizhou Medical Products Administration, Guiyang, China; 13 Department of Radiology, University of Washington School of Medicine, Seattle, WA USA; 14 Department of Pharmacology, Zhongshan School of Medicine, Sun Yat-Sen University, Guangzhou, China

**Keywords:** Cell death, Endocrine system and metabolic diseases

## Abstract

Ischemia/reperfusion (I/R)-induced liver injury with severe cell death is a major complication of liver transplantation. Transmembrane member 16A (TMEM16A), a component of hepatocyte Ca^2+^-activated chloride channel, has been implicated in a variety of liver diseases. However, its role in hepatic I/R injury remains unknown. Here, mice with hepatocyte-specific TMEM16A knockout or overexpression were generated to examine the effect of TMEM16A on hepatic I/R injury. TMEM16A expression increased in liver samples from patients and mice with I/R injury, which was correlated with liver damage progression. Hepatocyte-specific TMEM16A knockout alleviated I/R-induced liver damage in mice, ameliorating inflammation and ferroptotic cell death. However, mice with hepatic TMEM16A overexpression showed the opposite phenotype. In addition, TMEM16A ablation decreased inflammatory responses and ferroptosis in hepatocytes upon hypoxia/reoxygenation insult in vitro, whereas TMEM16A overexpression promoted the opposite effects. The ameliorating effects of TMEM16A knockout on hepatocyte inflammation and cell death were abolished by chemically induced ferroptosis, whereas chemical inhibition of ferroptosis reversed the potentiated role of TMEM16A in hepatocyte injury. Mechanistically, TMEM16A interacted with glutathione peroxidase 4 (GPX4) to induce its ubiquitination and degradation, thereby enhancing ferroptosis. Disruption of TMEM16A–GPX4 interaction abrogated the effects of TMEM16A on GPX4 ubiquitination, ferroptosis, and hepatic I/R injury. Our results demonstrate that TMEM16A exacerbates hepatic I/R injury by promoting GPX4-dependent ferroptosis. TMEM16A–GPX4 interaction and GPX4 ubiquitination are therefore indispensable for TMEM16A-regulated hepatic I/R injury, suggesting that blockades of TMEM16A–GPX4 interaction or TMEM16A inhibition in hepatocytes may represent promising therapeutic strategies for acute liver injury.

## Introduction

Ischemia–reperfusion (I/R) injury is an inevitable challenge after organ transplantation due to the technical nature of the surgery [1]. Severe hepatic I/R injury after liver transplantation leads to acute or chronic rejection and even graft failure by inducing inflammation and oxidative stress [2]. Therefore, understanding the pathogenesis of hepatic I/R injury is crucial for ensuring successful liver transplantation. Ferroptosis, a newly discovered form of cell death driven by iron- and lipid peroxidation-dependent regulation, is involved in various human diseases, thus creating considerable possibilities for ferroptosis-based therapeutic strategies [3]. A growing number of studies have reported that ferroptosis is implicated in hepatic I/R injury [4–6]. Ferritin-mediated iron overload in donor serum is considered a potential independent risk factor for hepatic I/R injury after liver transplantation [4]. Moreover, ferroptosis may be an essential trigger of hepatic I/R injury. Yamada et al. reported that treatment with the ferroptotic inhibitor ferrostatin-1 or the iron chelator deferoxamine inhibited liver damage and lipid peroxidation in a mouse model of hepatic I/R injury [4]. These results suggest that ferroptosis may provide a therapeutic target for hepatic I/R injury, although the underlying mechanisms of ferroptosis in hepatic I/R injury are not fully understood.

Transmembrane member 16A (TMEM16A) is involved in many important physiological and pathological functions, including inflammatory responses, oxidative stress, and cell survival or death [7–9]. Using a whole-cell patch clamp, researchers have identified TMEM16A as a Ca^2+^-activated chloride channel (CaCC) in guinea pigs and human hepatocytes derived from induced pluripotent stem cells [10, 11]. In our previous study, we observed similar conductance in mouse hepatocytes, and this chloride current was markedly potentiated by pro-steatotic challenge [9]. Further, TMEM16A was identified as a predominant chloride channel and highly expressed in mouse hepatocytes, promoting hepatic lipid accumulation, inflammation, and subsequent development of NAFLD [9]. These findings suggested that hepatocyte TMEM16A may play a critical role in regulating liver function. In addition, elevated TMEM16A expression and CaCC activation may be associated with pathologic phenotypes observed in cardiac and cerebral I/R mouse models [12, 13]. However, the involvement of TMEM16A in hepatic I/R injury remains poorly understood.

Notably, ferroptosis and the TMEM16A chloride channel are linked by complex molecular events [14, 15]. In multiple cell types, TMEM16A contributes to the production of excess reactive oxygen species (ROS) [8, 14], a key inducer of lipid peroxidation that leads to ferroptotic cell death. Reciprocally, lipid peroxidation activates TMEM16A and was shown to promote human and murine renal cyst enlargement, which was prevented by treatment with ferrostatin-1 or antioxidants [14]. Furthermore, inhibition of TMEM16A reduced lipid peroxidation and ferroptosis in mouse airway epithelial cells in response to bacterial infection [16]. Therefore, this study is aimed to investigate the functional role of hepatocyte TMEM16A in I/R-induced liver injury using mice with hepatocyte-specific TMEM16A knockout or overexpression. The results demonstrate that hepatocyte TMEM16A promotes hepatic I/R injury, whereas hepatocyte-specific knockout of TMEM16A alleviates acute liver injury by suppressing ferroptosis.

## Results

### TMEM16A is associated with hepatic I/R injury

**Fig. 1:**
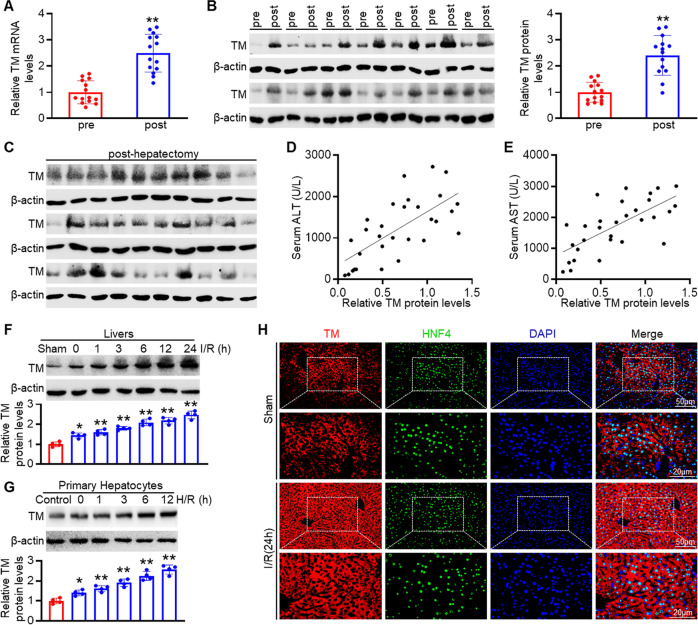
Hepatic TMEM16A expression is increased during I/R liver injury. Levels of TMEM16A (TM) **A** mRNA and **B** protein in liver samples harvested from patients pre- and post-hepatectomy, with β-actin as the inner control (*n* = 14). **P < 0.01 versus pre-hepatectomy. **C** Western blot analysis of TMEM16A protein expression in human liver samples obtained post-hepatectomy (*n* = 30). Pearson correlation analysis between post-hepatectomy hepatic TMEM16A protein expression and **D** serum ALT levels (*r*^2^ = 0.4701, *P* < 0.01) or **E** serum AST levels (*r*^2^ = 0.4821, *P* < 0.01). **F** Hepatic TMEM16A protein levels in mice challenged with the sham operation or 90 min of ischemia followed by reperfusion at the indicated times (*n* = 4). *P < 0.05, **P < 0.01 versus sham. **G** Western blot analysis of TMEM16A protein expression in primary hepatocytes subjected to 4 h of hypoxia followed by reoxygenation for the indicated times (*n* = 4). *P < 0.05, **P < 0.01 versus control. **H** Representative immunofluorescence of TMEM16A (red) and HNF4 (green) expression in liver sections of mice challenged with the sham operation or I/R (90 min of ischemia and 24 h of reperfusion) (*n* = 4). Nuclei were stained with DAPI (blue). Representative low- (upper images) and high-magnification (lower images) images are shown. Data were presented as the mean ± SD.

To explore the potential involvement of TMEM16A in hepatic I/R injury, TMEM16A expression was measured in liver samples obtained from patients pre- and post-hepatectomy. Both mRNA and protein levels of TMEM16A were significantly higher in post-hepatectomy samples than in pre-hepatectomy samples (Fig. 1A, B). Interestingly, the post-hepatectomy TMEM16A protein expression in patients was positively correlated with serum alanine aminotransferase (ALT) and aspartate aminotransferase (AST) levels (Fig. 1C–E). Furthermore, hepatic TMEM16A protein levels in mice gradually increased relative to sham group levels with increasing duration of reperfusion (Fig. 1F). Concurring with these results, enhanced TMEM16A expression was confirmed in primary hepatocytes challenged with hypoxia for 4 h followed by reoxygenation (Fig. 1G). Given that our previous study findings suggested that TMEM16A is an essential component of CaCC in hepatocytes [9], the relationship between CaCC current activation and increased TMEM16A expression upon H/R challenge was explored. The results indicated that the Ca^2+^-activated Cl^−^ current was markedly potentiated in primary hepatocytes after H/R insult (Fig. S1). Moreover, immunofluorescence staining for hepatocyte nuclear factor-4 (HNF4), a specific molecular marker of hepatocytes, revealed that TMEM16A expression was increased in hepatocytes in liver samples of mice after I/R injury (Fig. 1H). Collectively, these results suggested that amplified TMEM16A expression was involved in hepatic I/R injury.

### Hepatocyte-specific TMEM16A deficiency ameliorates hepatic I/R injury

**Fig. 2:**
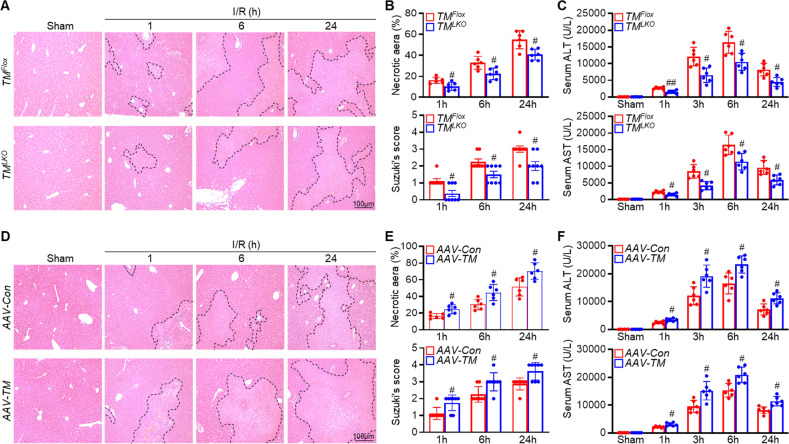
Hepatocyte-specific TMEM16A deficiency protects against hepatic I/R injury. **A** Representative H&E staining and **B** quantitation of necrotic areas (upper) and Suzuki histological scores (bottom) of liver tissues from *TM*^*Flox*^ and *TM*^*LKO*^ mice subjected to 90 min of hepatic ischemia followed by reperfusion for the indicated periods (*n* = 6–8). ^#^*P* < 0.05 versus corresponding *TM*^*Flox*^. **C** Serum ALT and AST levels in *TM*^*Flox*^ and *TM*^*LKO*^ mice from indicated groups (*n* = 6). ^#^*P* < 0.05, ^##^*P* < 0.01 versus corresponding *TM*^*Flox*^. **D** Representative H&E staining of liver sections from *AAV-Con-* or *AAV-TM*-treated mice subjected to sham operation or 90 min of hepatic ischemia followed by reperfusion for indicated periods. **E** Quantification of necrotic areas (upper) and Suzuki histological scores (bottom) (*n* = 6–8). ^#^*P* < 0.05 versus corresponding *AAV-Con*. **F** Serum ALT and AST levels in *AAV-Con-* and *AAV-TM-*treated mice from the indicated groups (*n* = 6). ^#^*P* < 0.05 versus corresponding *AAV-Con*. Data were presented as the mean ± SD.

The markedly increased hepatocyte TMEM16A levels after hepatic I/R injury prompted us to investigate the regulatory role of TMEM16A in this process. To this end, *TM*^*LKO*^ and *AAV-TM* mice were generated (Fig. S2A). TMEM16A knockout or overexpression in mouse livers was confirmed by western blotting (Fig. S2B, C). Histological examination of mouse liver sections revealed that the degree of liver damage gradually increased after 1 h of reperfusion. Compared with *TM*^*Flox*^ mice, *TM*^*LKO*^ mice displayed mitigated liver damage after I/R treatment, as shown by decreased necrotic areas and Suzuki scores (Fig. 2A, B). Additionally, serum ALT and AST levels were lower in *TM*^*LKO*^ mice than in *TM*^*Flox*^ mice (Fig. 2C). In contrast, *AAV-TM*-treated mice exhibited more severe liver injury, along with increased necrotic areas and Suzuki scores, than *AAV-Con*-treated mice (Fig. 2D, E). Moreover, *AAV-TM*-treated mice displayed higher serum ALT and AST levels than *AAV-Con*-treated mice (Fig. 2F). These observations indicated that TMEM16A promoted the development of hepatic I/R injury.

### TMEM16A knockout in hepatocytes suppresses inflammation during hepatic I/R injury

**Fig. 3:**
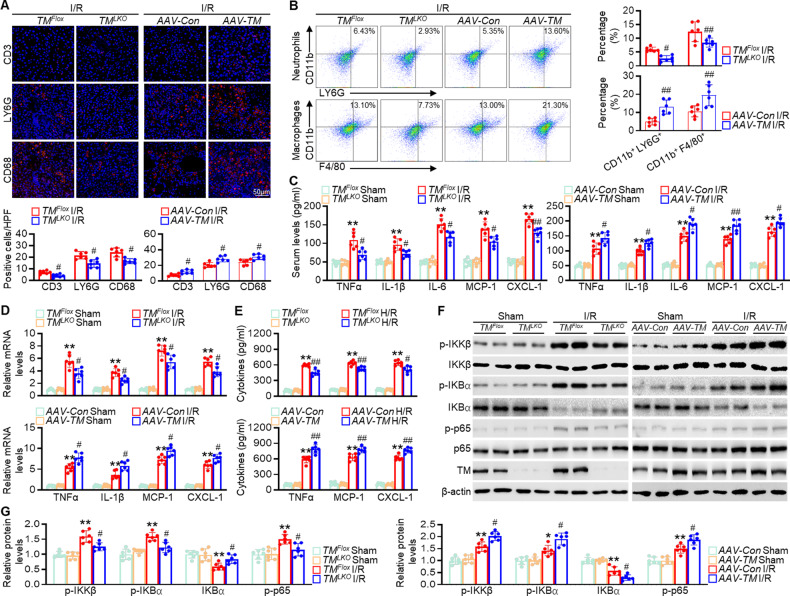
Hepatocyte-specific TMEM16A deficiency ameliorates inflammation during hepatic I/R injury. **A** Representative immunofluorescence staining and quantification of infiltrating CD3^+^, LY6G^+^, or CD68^+^ cells (red) in the livers of *TM*^*LKO*^ or *AAV-TM* mice and their counterpart control mice subjected to 90 min of hepatic ischemia followed by 24 h of reperfusion (*n* = 6). ^#^*P* < 0.05 versus *TM*^*Fl*ox^ or *AAV-Con*. **B** Flow cytometry histogram showing neutrophils and macrophages in liver samples from indicated mice subjected to 90 min of ischemia and 24 h of reperfusion (*n* = 6). ^#^*P* < 0.05, ##P < 0.01 versus *TM*^*Flox*^ I/R or *AAV-Con* I/R. Levels of **C** serum and **D** hepatic mRNA levels of pro-inflammatory factors (TNF-α, IL-1β, Il-6, MCP-1, and CXCL-1) in *TM*^*LKO*^ or *AAV-TM-*treated mice and their counterparts subjected to 90 min of hepatic ischemia followed by 24 h of reperfusion (*n* = 6). **P < 0.01 versus *TM*^*Flox*^ sham or *AAV-Con* sham; ^#^*P* < 0.05, ^##^*P* < 0.01 versus *TM*^*Flox*^ I/R or *AAV-Con* I/R. **E** TNF-α, MCP-1, and CXCL-1 levels in hepatocytes isolated from *TM*^*LKO*^ or *AAV-TM*-treated mice and their counterpart control mice after H/R challenge (4 h of hypoxia and 12 h of reoxygenation) (*n* = 6). **P < 0.01 versus *TM*^*Flo*^ or *AAV-Con*; ^#^*P* < 0.05, ^##^*P* < 0.01 versus *TM*^*Flox*^ H/R or *AAV-Con* H/R. F Total and phosphorylated IKKβ, IκBα, and p65 levels in livers of *TM*^*LKO*^ or *AAV-TM*-treated mice after sham operation or hepatic I/R treatment (90 min of ischemia and 24 h of reperfusion) (*n* = 6). **G** Relative levels of the aforementioned proteins. *P < 0.05, **P < 0.01 versus *TM*^*Flox*^ sham or *AAV-Con* sham; #*P* < 0.05 versus *TM*^*Flox*^ I/R or *AAV-Con* I/R. Data were presented as the mean ± SD.

Given the critical role of the inflammatory response in hepatic I/R injury [17] and the potential regulation of hepatic I/R injury by TMEM16A, we explored the relationship between TMEM16A and inflammation during I/R-induced liver injury. Immunofluorescence staining for *CD3*^*+*^ T cells, *LY6G*^*+*^ neutrophils, and *CD68*^*+*^ macrophages revealed that the numbers of inflammatory cells after hepatic I/R challenge were significantly lower in *TM*^*LKO*^ mouse livers than in *TM*^*Flox*^ mouse livers. However, hepatocyte-specific TMEM16A overexpression had the opposite effect on inflammatory cell infiltration (Fig. 3A). Similarly, flow cytometry analysis of *LY6G*^*+*^*CD11b*^*+*^ and *F4/80*^*+*^*CD11b*^*+*^ cells collected from NPCs further confirmed that numbers of inflammatory cells after hepatic I/R injury were lower in *TM*^*LKO*^ mice but higher in *AAV-TM-*treated mice than in corresponding controls (Fig. 3B). No significant inflammatory cell infiltration was observed in sham mice (Fig. S3A, B). Moreover, serum and hepatic mRNA levels of inflammatory cytokines (TNF-α and IL-1β) and chemokines (C-X-C motif chemokine ligand 1 [CXCL-1] and monocyte chemoattractant protein-1 [MCP-1]) were inhibited in *TM*^*LKO*^ mice after hepatic I/R injury but potentiated in *AAV-TM*-treated mice (Fig. 3C, D).

Consistent with the results of the in vivo experiments, TNF-α, MCP-1, and CXCL-1 levels were markedly lower in primary hepatocytes isolated from *TM*^*LKO*^ mice upon H/R treatment than in those isolated from *TM*^*Flox*^ mice, whereas TMEM16A overexpression was associated with increased inflammatory cytokine and chemokine secretion (Fig. 3E). In addition, canonical NF-κB signaling was activated in mouse livers after I/R treatment and in hepatocytes after H/R challenge, as shown by elevated phosphorylation of IκB kinase β (IKKβ), an inhibitory subunit of NF-κBα (IκBα), and p65, as well as degradation of IκBα. This signaling activation was blocked by TMEM16A knockout but further enhanced by TMEM16A overexpression (Fig. 3F, G; Fig. S4A, B). Collectively, these findings suggested that hepatocyte TMEM16A exacerbated I/R-induced hepatic inflammation.

### TMEM16A deletion inhibits hepatic I/R-induced ferroptosis

**Fig. 4:**
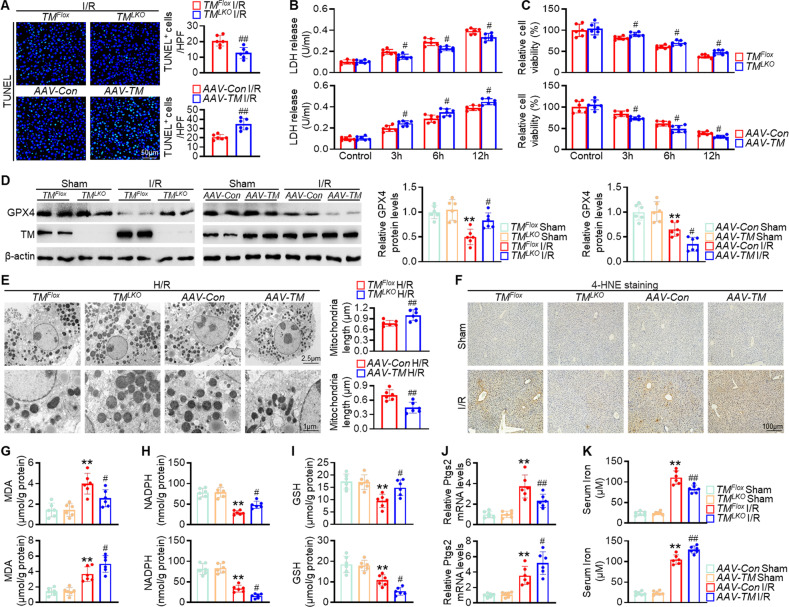
TMEM16A deletion protects against hepatic I/R-induced ferroptosis. **A** Representative TUNEL immunofluorescence staining and quantification of TUNEL-positive cells in liver sections from *TM*^*Flox*^, *TM*^*LKO*^, *AAV-Con*-treated, and *AAV-TM*-treated mice subjected to 90 min of hepatic ischemia followed by 24 h of reperfusion (*n* = 6). ^##^*P* < 0.01 versus *TM*^*Flox*^ I/R or *AAV-Con* I/R. **B** LDH release and **C** CCK-8 assay results of hepatocytes isolated from *TM*^*LKO*^ mice or *AAV-TM*-treated mice and their counterpart control mice subjected to 4 h of hypoxia followed by reoxygenation (*n* = 6). ^#^*P* < 0.05 versus corresponding *TM*^*Flox*^ or *AAV-Con*. **D** Hepatic GPX4 protein levels in livers of mice challenged with the sham operation or 90 min of ischemia followed by 24 h of reperfusion (*n* = 6). **P < 0.01 versus *TM*^*Flox*^ sham or *AAV-Con* sham; ^#^*P* < 0.05 versus *TM*^*Flox*^ I/R or *AAV-Con* I/R. **E** Representative transmission electron microscopy images and quantification of mitochondria length in primary hepatocytes isolated from indicated groups after 4 h of hypoxia and 12 h of reoxygenation (*n* = 6). ^##^*P* < 0.01 versus *TM*^*Flox*^ H/R or *AAV-Con* H/R. **F** Representative images of 4-HNE immunohistochemical staining in liver samples from mice subjected to 90 min of hepatic ischemia followed by 24 h of reperfusion (*n* = 6). Hepatic mRNA levels of **G** MDA, **H** NADPH, **I** GSH, and **J** PTGS2, as well as **K** serum iron content in indicated groups (*n* = 6). **P < 0.01 versus *TM*^*Flox*^ sham or *AAV-Con* sham; ^#^*P* < 0.05, ^##^*P* < 0.01 versus *TM*^*Flox*^ I/R or *AAV-Con* I/R. Data were presented as the mean ± SD.

Cell death is a direct contributor to liver damage during acute liver injury [4, 5, 18]. TUNEL staining of liver sections revealed that hepatic I/R-induced cell death was suppressed in *TM*^*LKO*^ mice but potentiated in *AAV-TM*-treated mice compared with that in corresponding control mice (Fig. 4A). The abundance of TUNEL-positive cells remained consistent in mice with either TMEM16A deletion or overexpression that underwent a sham operation (Fig. S5). The effects of TMEM16A on hepatocyte survival were further confirmed by the results of the LDH release and cell viability assays. Consistently, hepatocytes isolated from *TM*^*LKO*^ mice showed lower LDH levels and higher viability upon H/R insult than those isolated from *TM*^*Flox*^ mice. The opposite effects were observed in hepatocytes isolated from mice overexpressing TMEM16A (Fig. 4B, C).

To investigate the precise role of TMEM16A in cell death regulation, its effects on apoptosis were explored. Cleaved caspase-3 levels were similar between *TM*^*LKO*^ and *TM*^*Flox*^ mouse livers upon hepatic I/R injury (Fig. S6A). Additionally, levels of phosphorylated mixed lineage kinase domain-like pseudokinase (MLKL), as well as NLR family pyrin domain containing 3 (NLRP3) and cleaved caspase-1, the hallmarks of necroptosis and pyroptosis, respectively, were comparable between *TM*^*LKO*^ and *TM*^*Flox*^ mouse livers after hepatic I/R injury (Fig. S6B, C). Interestingly, expression of GPX4, a central negative regulator of ferroptosis [3, 19], was markedly higher in *TM*^*LKO*^ mouse livers than in *TM*^*Flox*^ mouse livers in response to I/R. However, TMEM16A overexpression was associated with decreased GPX4 expression (Fig. 4D), indicating that TMEM16A acted as a negative regulator of GPX4 and that ferroptosis was involved in the effects of TMEM16A on cell death during acute liver injury. Moreover, GPX4 expression was negatively correlated with TMEM16A expression in patients post-hepatectomy (Fig. S7). To confirm the involvement of ferroptosis in this process, ultrastructural analysis of primary hepatocytes was performed using TEM. TMEM16A knockout or overexpression had no effect on the mitochondrial morphology of hepatocytes under basal conditions (Fig. S8). However, H/R treatment led to shrunken mitochondria with increased membrane density, which was less pronounced in hepatocytes isolated from *TM*^*LKO*^ mice after H/R treatment but further reinforced in those isolated from *AAV-TM* mice (Fig. 4E). Furthermore, H/R-induced accumulation of lipid ROS, a major pathological event in ferroptosis [3], was markedly blunted in hepatocytes isolated from TMEM16A knockout mice. Conversely, hepatocytes isolated from *AAV-TM*–treated mice showed increased lipid ROS accumulation (Fig. S9). Concurring with the in vitro results, *TM*^*LKO*^ mice with hepatic I/R injury exhibited significantly inhibited hepatic lipid peroxidation along with decreased IHC staining for 4-hydroxy-2-nonenal (4-HNE) and MDA levels, whereas *AAV-TM*-treated mice displayed elevated hepatic lipid peroxidation levels (Fig. 4F, G). Additionally, NADPH and GSH levels were remarkably higher in *TM*^*LKO*^ mouse livers upon hepatic I/R injury than in *TM*^*Flox*^ mouse livers, whereas hepatic mRNA expression of prostaglandin-endoperoxide synthase 2 (PTGS2) and serum iron levels were lower. In contrast, *AAV-TM*-treated mice exhibited the opposite effects (Fig. 4H–K). Taken together, these findings suggested that TMEM16A was an important regulator of ferroptosis during hepatic I/R injury.

### GPX4-dependent ferroptosis is essential for TMEM16A-mediated exacerbation of hepatic I/R injury

**Fig. 5:**
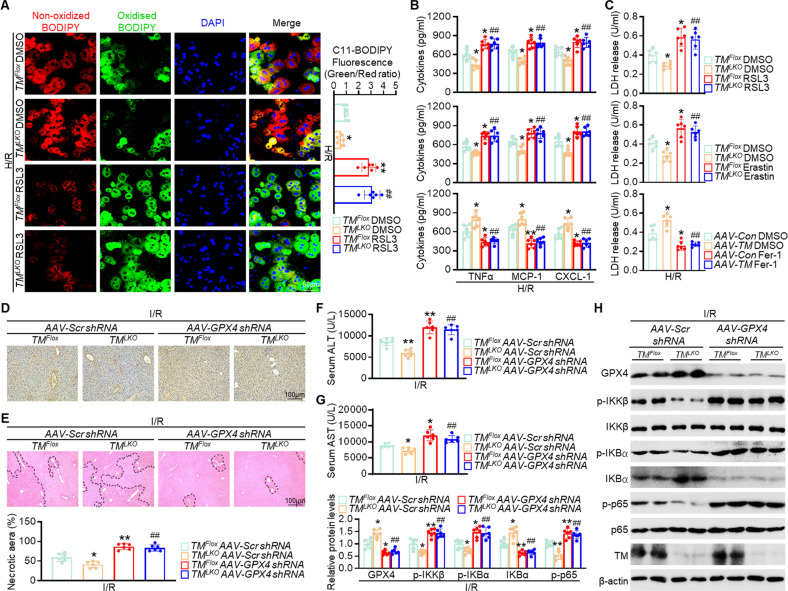
TMEM16A regulates hepatic I/R injury in a GPX4-dependent manner. **A** Lipid ROS levels of primary hepatocytes isolated from indicated mice treated with RSL3 (3 μmoL/L) for 8 h followed by 4 h of hypoxia and 12 h of reoxygenation (*n* = 6). Red, reduced form of C11-BODIPY; a green, oxidized form of C11-BODIPY. *P < 0.05, **P < 0.01 versus *TM*^*Flox*^ DMSO; ^##^*P* < 0.01 versus *TM*^*LKO*^ DMSO. **B** Levels of pro-inflammatory factors (TNF-α, MCP-1, and CXCL-1) and **C** LDH release in indicated hepatocytes treated with RSL3, Erastin (10 μmoL/L), or Ferrostatin-1 (Fer, 2 μmoL/L) for 8 h followed by 4 h of hypoxia and 12 h of reoxygenation (*n* = 6). *P < 0.05, **P < 0.01 versus *TM*^*Flox*^ DMSO or *AAV-Con* DMSO; ^##^*P* < 0.01 versus *TM*^*LKO*^ DMSO or *AAV-TM* DMSO. **D** Representative 4-HNE immunohistochemical staining of liver samples from *TM*^*LKO*^ and *TM*^*Flox*^ mice injected with *AAV-GPX4 shRNA* via tail vein two weeks prior to I/R surgery (*n* = 6). **E** Representative H&E staining of liver sections, necrotic area quantification (area outside the contour indicates the injured part in the last two pictures on the right side), and **F** serum ALT and **G** AST levels in indicated mice (*n* = 6). *P < 0.05, **P < 0.01 versus *TM*^*Flox*^ *AAV-Scr shRNA*; ^##^*P* < 0.01 versus *TM*^*LKO*^ *AAV-Scr shRNA*. **H** Expression of proteins associated with the NF-κB signaling pathway in liver samples from indicated mice (*n* = 6). c versus *TM*^*Flox*^ *AAV-Scr shRNA*; ^##^*P* < 0.01 versus *TM*^*LKO*^ *AAV-Scr shRNA*. Data were presented as the mean ± SD.

To verify the role of ferroptosis in TMEM16A-mediated hepatic I/R injury, primary hepatocytes isolated from *TM*^*LKO*^ and *AAV-TM*-treated mice were treated with either ferroptotic inducers (RSL3 or Erastin) or a ferroptotic inhibitor (ferrostatin-1), followed by H/R challenge. The inhibitory effects of TMEM16A deficiency on lipid ROS levels in hepatocytes were markedly abolished by pharmacological induction of ferroptosis. On the other hand, pharmacological inhibition of ferroptosis resulted in decreased lipid ROS levels in hepatocytes isolated from either *AAV-TM*- or *AAV-Con*-treated mice (Fig. 5A; Fig. S10A, B). Furthermore, the administration of ferroptotic inducers reversed this inhibition of cell death and the inflammatory response observed in hepatocytes isolated from *TM*^*LKO*^ mice upon H/R insult. However, treatment with the ferroptotic inhibitor ameliorated H/R-induced cell death and inflammation, showing a comparable effect to that seen between hepatocytes isolated from *AAV-Con-* and *AAV-TM*-treated mice and contrasting with vehicle-treated cells (Fig. 5B, C; Fig. S10C, D). These findings suggested that ferroptosis was responsible for the regulatory function of TMEM16A in hepatic I/R-related pathological events.

The importance of GPX4 in ferroptosis and reduced GPX4 expression in mice overexpressing hepatic TMEM16A suggested that GPX4 could be the key factor in regulating TMEM16A-mediated ferroptosis and hepatic liver injury. Therefore, *AAV-GPX4 shRNA* or scrambled shRNA (*AAV-Scr shRNA*) was administered to *TM*^*LKO*^ and *TM*^*Flox*^ mice via tail vein injection two weeks prior to I/R treatment. The efficiency of GPX4 knockdown in the liver was verified by western blotting (Fig. S11A). No significant differences were observed in lipid peroxidation or necrotic area among the groups under sham conditions (Fig. S12A, B). However, *AAV-GPX4 shRNA* administration significantly potentiated the I/R-induced increase in hepatic lipid peroxidation levels, PTGS2 mRNA expression, and serum iron content. Moreover, GPX4 knockdown abrogated the inhibition of these ferroptotic events in mice with hepatocyte-specific TMEM16A deficiency (Fig. 5D; Fig. S11B, C). Furthermore, GPX4 knockdown abolished the protective effects of TMEM16A deficiency on liver damage, as shown by increased necrotic area, serum ALT and AST levels, and hepatic inflammatory response (Fig. 5E-H). These results indicated that GPX4-meditated ferroptosis contributed to the deleterious effects of TMEM16A in hepatic I/R injury.

### TMEM16A directly interacts with GPX4 and contributes to its degradation

**Fig. 6:**
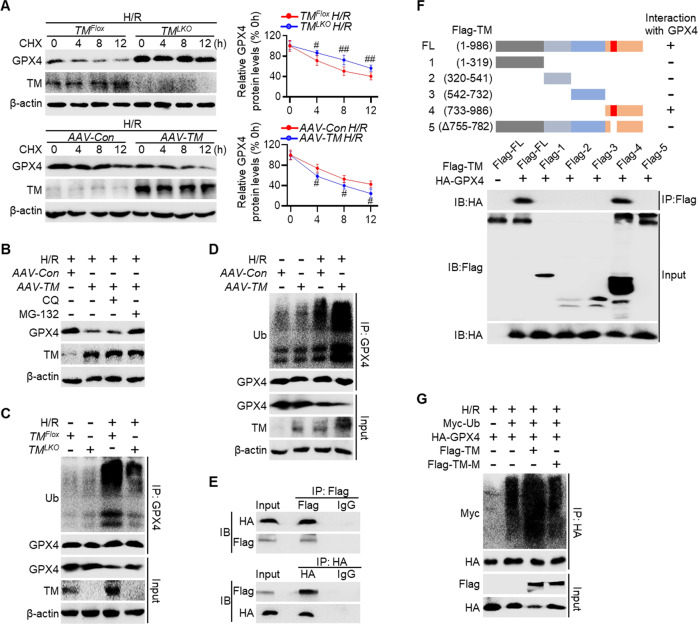
TMEM16A directly binds with GPX4 and facilitates its ubiquitination. **A** Western blotting results of GPX4 protein expression in hepatocytes treated with 4 h of hypoxia and 12 h of reoxygenation, followed by incubation with cycloheximide (CHX, 100 µg/mL) for the indicated durations (*n* = 6). ^#^*P* < 0.05, ^##^*P* < 0.01 versus *TM*^*Flox*^ H/R or *AAV-Con* H/R. **B** GPX4 protein expression in hepatocytes isolated from *AAV-TM*-treated mice after 4 h of hypoxia and 12 h of reoxygenation, followed by treatment with MG-132 (10 µmol/L) or chloroquine (CQ, 20 µmol/L) for 4 h (*n* = 6). GPX4 protein expression in hepatocytes isolated from **C** *TM*^*LKO*^ and **D** *AAV-TM*-treated mice and their control littermates after 4 h of hypoxia and 12 h of reoxygenation (n = 4). Immunoprecipitation was performed with an anti-GPX4 antibody followed by western blot analysis of GPX4 ubiquitination. **E** Flag and HA expression in HEK293T cells co-transfected with vectors expressing Flag-TMEM16A and HA-GPX4 (n = 6). Immunoprecipitation (IP) was performed with an anti-Flag antibody (upper panel) or anti-HA antibody (lower panel), followed by western blotting with an anti-HA antibody or anti-Flag antibody. **F** Results of IP assay showing interaction domains of TMEM16A and GPX4 determined using full-length and truncated TMEM16A expression constructs (*n* = 4). **G** Results of IP assay showing GPX4 ubiquitination in L02 cells transfected with HA-GPX4, in combination with Myc-Ubiquitin, Flag-TMEM16A, and Flag-TM-M, followed by H/R treatment (*n* = 4). Data were presented as the mean ± SD.

Next, the effects of TMEM16A regulation on GPX4 expression were investigated. Notably, the I/R-induced decrease in hepatic GPX4 mRNA levels was not altered by TMEM16A knockout or overexpression in vivo (Fig. S13A, B), suggesting post-translational regulation of gene expression. Treatment of hepatocytes with an inhibitor of protein synthesis, cycloheximide, led to a time-dependent decrease in GPX4 protein expression. The decline in GPX4 protein expression in hepatocytes isolated from TMEM16A knockout mice was inhibited, but this decline was enhanced in hepatocytes isolated from TMEM16A-overexpressing mice (Fig. 6A), indicating an essential role of TMEM16A in GPX4 degradation. To elucidate the molecular mechanism underlying GPX4 degradation, hepatocytes isolated from TMEM16A-overexpressing mice were treated with either a lysosomal inhibitor (chloroquine) or a proteasomal inhibitor (MG-132), as lysosome- and proteasome-dependent degradation pathways are the primary contributors to protein degradation [20]. MG-132 treatment nearly restored GPX4 expression, whereas chloroquine treatment had a negligible effect (Fig. 6B). These results suggested that GPX4 degradation occurred via the proteasomal pathway, in which ubiquitination is necessary for protein modification [21]. Indeed, ubiquitination of GPX4 was considerably induced by H/R challenge, and this effect was abolished when TMEM16A was knocked out but augmented when TMEM16A was overexpressed (Fig. 6C, D).

The molecular link between TMEM16A and GPX4 was further investigated via IP assay, revealing through exogenous transfection with TMEM16A-flag and GPX4-HA that TMEM16A bound to GPX4 (Fig. 6E). A series of truncated fragments of Flag-TMEM16A were generated to clarify which TMEM16A domain was required for the interaction between TMEM16A and GPX4. The results indicated that GPX4 interacted with the C-terminal region of TMEM16A, spanning amino acids (aa) 733–986, as well as full-length TMEM16A (Fig. 6F). Considering the cytoplasmic distribution of GPX4 and the multidomain, transmembrane nature of TMEM16A [22], the GPX4-binding region of TMEM16A was speculated to be located in the cytoplasm. Using the Prediction of Transmembrane Helices in Proteins tool (https://services.healthtech.dtu.dk/service.php?TMHMM-2.0), residues 755–782 within the 733–986 aa region of TMEM16A were predicted to be responsible for binding to GPX4. Indeed, a mutation in this region (755–782 aa) abolished the interaction between TMEM16A and GPX4 (Fig. 6F). Furthermore, a mutation in this region eliminated TMEM16A-induced ubiquitination of GPX4 after H/R treatment (Fig. 6G). Collectively, the data demonstrated that TMEM16A bound to GPX4 and promoted its degradation.

### The TMEM16A domain responsible for GPX4 binding is indispensable to TMEM16A function in hepatic I/R injury

**Fig. 7:**
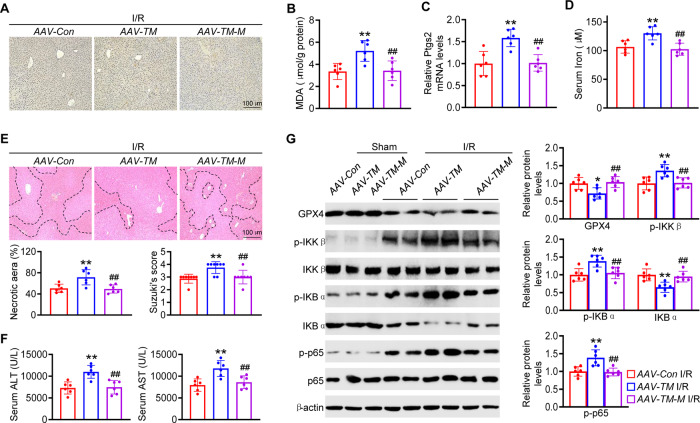
The interaction between TMEM16A and GPX4 is indispensable for TMEM16A function in hepatic I/R injury. **A** Representative immunohistochemical staining of 4-HNE in liver sections of *AAV-Con-*, *AAV-TM-*, and *AAV-TM-M-*treated mice subjected to 90 min of hepatic ischemia followed by 24 h of reperfusion. Hepatic levels of **B** MDA, **C** PTGS2, and **D** serum iron content (*n* = 6). **P < 0.01 versus *AAV-Con* I/R; ^##^*P* < 0.01 versus *AAV-TM* I/R. **E** Representative H&E staining, necrotic area quantification, and Suzuki histological score in liver samples, and **F** serum ALT and AST levels in indicated mice followed by 90 min of ischemia and 24 h of reperfusion (*n* = 6–8). **P < 0.01 versus *AAV-Con* I/R; ^##^*P* < 0.01 versus *AAV-TM* I/R. **G** Western blot analysis and quantification of levels of hepatic GPX4 protein and proteins related to the NF-κB signaling pathway from the indicated groups (*n* = 6). *P < 0.05, **P < 0.01 versus *AAV-Con* I/R; ^##^*P* < 0.01 versus *AAV-TM* I/R. Data were presented as the mean ± SD.

To evaluate whether an interaction between TMEM16A and GPX4 is necessary for the regulatory function of TMEM16A in hepatic I/R injury, wild-type mice were administered an adenovirus harboring TMEM16A with a mutant 755–782 aa domain (*AAV-TM-M*) via tail vein injection. TMEM16A-overexpressing mice exhibited clear pro-ferroptotic effects after hepatic I/R challenge for 24 h, including elevated lipid peroxidation levels, hepatic PTGS2 mRNA expression, and serum iron content. *AAV-TM-M* treatment yielded comparable effects on ferroptosis to those of *AAV-Con* treatment (Fig. 7A–D). Moreover, the increased degree of liver damage observed in *AAV-TM*-treated mice was significantly reversed in *AAV-TM-M*-treated mice, as evidenced by decreased necrotic area, Suzuki scores, and serum ALT and AST levels (Fig. 7E, F). In addition, western blotting revealed that the capacity of TMEM16A to promote the inflammatory response was abrogated by the blockade of TMEM16A-GPX4 interaction (Fig. 7G). Taken together, the results suggested that TMEM16A-GPX4 interaction was indispensable for the function of TMEM16A in hepatic I/R injury.

## Discussion

The present study provides evidence that TMEM16A expression increased in patients and mice with I/R injury. Furthermore, hepatocyte-specific TMEM16A knockout dramatically attenuated the hepatic inflammatory response and ferroptosis in mice upon I/R injury, whereas hepatocyte-specific TMEM16A overexpression produced the opposite results. Further investigation revealed that TMEM16A directly interacted with GPX4 and mediated its ubiquitination and degradation in response to I/R challenge. Importantly, inhibiting the regulatory axis composed by TMEM16A and GPX4 almost completely reversed the potentiated effects of TMEM16A in I/R-induced injury, suggesting TMEM16A as a promising therapeutic target for hepatic I/R injury.

Emerging studies have revealed that CaCC, encoded by *TMEM16A*, is involved in several I/R-induced pathological events, such as myocardial ischemia, arrhythmia, and stroke [12, 13, 23]. Moreover, its expression has been suggested to be upregulated in many diseases, including hypertension, diabetes, cystic fibrosis, and cancer [8, 24–26]. Our previous study demonstrated that TMEM16A was an essential component of CaCC in hepatocytes and increased during nonalcoholic fatty liver disease [9], indicating the involvement of TMEM16A in liver-related disease. However, the potential role of TMEM16A in I/R-induced liver damage has not been elucidated. Here, we provide clear evidence that hepatic TMEM16A was upregulated in response to I/R challenge and that deletion of TMEM16A produced beneficial effects against hepatic I/R injury, but the mechanism by which I/R induces TMEM16A expression in the liver remains to be addressed. The mechanisms underlying TMEM16A upregulation include transcriptional and post-transcriptional gene regulation [27–29]. Moreover, DNA methylation and microRNA (e.g., miR-9 and miR-144-3p) were recently proposed to be important regulators of TMEM16A expression [30–33]. Furthermore, epigenetic reprogramming has also been found to regulate various genes during I/R injury [32, 34]. For example, miR-9 restrained TMEM16A expression in a canonical manner [31], and upregulation of miR-9 was able to ameliorate hepatic I/R injury [32]. However, it remains to be determined whether these potential factors are directly involved in hepatic I/R-regulated TMEM16A expression.

Complicated crosstalk occurs between TMEM16A and cell death [14, 16, 35]. Previous studies have reported that TMEM16A promotes DNA damage-induced apoptosis via activation of the caspase cascade [36–38]. However, in the present study, neither overexpression nor deficiency of TMEM16A altered the expression of cleaved caspase-3, suggesting that apoptosis was not involved in TMEM16-mediated hepatocyte death during acute liver injury. Different outcomes related to cell death may be associated with variations in cell type, tissue location, and pathological conditions. Additionally, the effects of TMEM16A on I/R-induced hepatocyte death were not associated with the regulation of necroptosis and pyroptosis. Therefore, we hypothesize that TMEM16A may be involved in other forms of cell death. Hepatic I/R injury was accompanied by iron overload and lipid peroxidation, consequently contributing to ferroptosis. These findings concur with reports that the I/R challenge can induce ferroptosis in the heart, kidneys, and lungs [39–41]. Furthermore, TMEM16A activation has been associated with oxidative lipid damage [8, 14]. The current study provides evidence that hepatocyte-specific knockout of TMEM16A ameliorates ferroptotic cell death, suggesting that TMEM16A functions as an inducer of ferroptosis in acute liver injury. More importantly, pharmacological inhibition of ferroptosis largely reversed the enhanced effects of TMEM16A in hepatic I/R injury, aligning with the results of a previous study in which liver damage after I/R challenge was ameliorated by ferroptotic inhibitor treatment [4]. Therefore, our findings demonstrate that TMEM16A potentiates I/R-induced liver damage by enhancing ferroptosis in hepatocytes.

TMEM16A is reportedly upregulated or activated by a range of inflammatory mediators (e.g., IL-4 and IL-6) and subsequently plays a key role in the pathology of various inflammation-related diseases [7, 9, 38], suggesting a potential relationship between TMEM16A and inflammation. I/R-induced inflammation is an important trigger for acute liver damage [17, 18, 42]. The accompanying infiltration of inflammatory cells, including T cells, neutrophils, and macrophages, contributes to hepatic I/R injury via diverse mechanisms [43–45]. Moreover, NF-κB signaling has been documented to be a critical molecular event in the progression of hepatic I/R injury [17, 42]. In the current study, activation of NF-κB signaling was indeed involved in TMEM16A-mediated exacerbation of hepatic I/R injury, as reflected by increased cytokine production and inflammatory cell infiltration. This observation aligned with our previously reported results whereby TMEM16A promoted NF-κB signaling and inflammation under steatohepatitis conditions [9]. Notably, emerging evidence indicates an interplay between ferroptosis and inflammation. In nonalcoholic steatohepatitis (NASH), ferroptosis triggers the infiltration of immune cells and inflammatory response, promoting disease progression [6]. Inhibition of ferroptosis effectively reversed hepatic inflammation and liver dysfunction induced by the environmental toxin ethyl carbamate. Furthermore, a recent study reported that the nonapoptotic form of I/R-related cell death induced by ferroptosis triggered activation of the innate immune system, which is thought to be necessary for the initiation of inflammation [46]. In the present study, treatment with a ferroptotic inhibitor prevented activation of TMEM16A-mediated NF-κB signaling and inflammatory response. Taken together, the results suggest that TMEM16A-mediated ferroptosis is an upstream regulator of inflammation during hepatic I/R injury.

GPX4 is the cornerstone of anti-ferroptotic defense due to its ability to reduce phospholipid hydroperoxides to lipid alcohols [3, 19]. Among GPX isoforms, GPX4 is the only enzyme capable of reducing phospholipid hydroperoxides in membranes. GPX4 exerts its unique and important function in murine embryogenesis, as evidenced by embryonic lethality in conventional whole-body GPX4 knockout mice, which is not observed when other GPX genes are systemically knocked out [19, 47]. Further, studies have reported lipid peroxidation upon conditional deletion of GPX4, highlighting GPX4 inactivation as the key executor of lipid peroxide formation, which ultimately leads to ferroptosis [39, 48]. Moreover, programmed cell death controlled by the GPX4-dependent signaling cascade was shown to be necessary for the pathological process of I/R-induced injury in the liver and other organs [39, 49]. Nevertheless, the current study is the first to suggest that TMEM16A is a key regulator of GPX4 during hepatic I/R injury. Overexpression of TMEM16A decreased hepatic GPX4 expression, whereas TMEM16A knockout induced the opposite effect. More importantly, GPX4 knockdown reversed the inhibition of ferroptosis in TMEM16A knockout mice, subsequently abolishing the protective effects against liver injury. It should be noted that the hepatocyte-specific GPX4 knockdown mice used in the current study were developmentally normal, unlike the hepatocyte GPX4 knockdown mice used in a previous study that died 24–48 h after birth due to severe liver dysfunction [48]. These results further confirm the indispensable role of GPX4 in regulating physiological events, suggesting that sustaining GPX4 expression within a limited range is a more promising strategy than complete knockout to maintain the functional homeostasis of the liver.

Notably, GPX4 protein expression was affected by TMEM16A during I/R injury, but GPX4 mRNA levels were not altered. TMEM16A-dependent degradation of GPX4 protein was confirmed by the results of the protein stability assay. Recent studies reported that chaperone-mediated autophagy, a cellular lysosome-mediated degradation mechanism, is involved in mediating GPX4 degradation by binding HSC70 and HSP90 [50, 51]. However, treatment with a lysosomal inhibitor failed to reverse TMEM16A-induced suppression of GPX4 protein levels upon H/R insult in the current study. Alternatively, studies have suggested that GPX4 degradation also involves ubiquitin modification [52–54]. Concurrent with these findings, treatment with a proteasomal inhibitor rescued the reduced GPX4 protein levels, suggesting that the ubiquitin–proteasome pathway is at least partially responsible for GPX4 degradation. Indeed, TMEM16A is directly bound to GPX4 and contributed to its ubiquitination. Although the study findings suggest that TMEM16A functions as an adapter to recruit E3 ubiquitin ligases to attach a ubiquitin chain to GPX4, further studies are warranted to determine which E3 ligase(s) is (are) required for TMEM16A-mediated, proteasome-dependent degradation of GPX4. The results of the present study also suggest that the TMEM16A fragment comprising residues 755–782 is essential for interaction with GPX4. Further, truncated TMEM16A lacking the GPX4-binding domain failed to promote GPX4 ubiquitination and degradation, producing no effects on hepatocyte ferroptosis and hepatic I/R injury. Therefore, this study elucidated the molecular mechanism by which TMEM16A interacts with GPX4, which is indispensable for the regulatory role of TMEM16A in hepatic I/R injury. Further structural and functional analysis of the TMEM16A sequence that controls binding with GPX4 (755–782 aa) may open potential options for modulating GPX4 interactions and ferroptosis-related disease treatment.

In conclusion, the current study highlights TMEM16A as a prominent initiator of hepatic I/R injury. TMEM16A directly interacts with GPX4 and facilitates its degradation through the ubiquitin–proteasome pathway, leading to ferroptosis and hepatic I/R injury. Therefore, targeting TMEM16A or blocking the TMEM16A–GPX4 axis may represent promising approaches to prevent or treat hepatic I/R injury.

## Materials and methods

### Human liver samples

All procedures involving humans were performed in accordance with the Human Ethics Committee of Zhujiang Hospital of Southern Medical University (approval no. 2022-KY-055-01), Jinzhou Central Hospital (approval no. 2022-032-01), and the Declaration of Helsinki. Written informed consent was obtained from all participants.

Non-fatty liver biopsy specimens were obtained from 30 patients diagnosed with benign liver disease who underwent hepatectomy. Pre-hepatectomy liver biopsy specimens were harvested prior to hepatic portal occlusion, and post-hepatectomy specimens were collected about 30 min after portal reperfusion (prior to abdominal closure). Ischemic time ranged from 15 to 20 min. All biopsy specimens were immediately frozen in liquid nitrogen for later use. Blood samples for liver enzyme analysis were obtained 1 d after liver resection. The clinical characteristics of patients are summarized in Table S1.

### Reagents

RSL3, Erastin, and ferrostatin-1 were obtained from Selleck (Shanghai, China). Unless otherwise indicated, all reagents were purchased from Sigma-Aldrich (St. Louis, MO, USA).

### Animals

TMEM16A-floxed homozygous mice (*TM*^*Flox*^) were generated by Cyagen (Suzhou, China) as previously described [9]. Briefly, TMEM16A exon 12 was floxed with two loxP sites on the C57BL/6J background. *Alb-Cre* mice (The Jackson Laboratory, Bar Harbor, ME, USA) were mated with *TM*^*Flox*^ mice. Hepatocyte-specific TMEM16A knockout mice (*TM*^*LKO*^) were generated by self-mating the offspring. The PCR primers for genotyping these mice are listed in Supporting Table S2.

In terms of generating mice with hepatocyte-specific TMEM16A overexpression (*AAV-TM*), the full-length TMEM16A sequence was cloned into an adeno-associated virus serotype 8 (AAV8) vector containing the thyroxine-binding globulin (AAV8-TBG) promoter, which enables the specific expression of target genes in hepatocytes. Additionally, the TMEM16A mutant sequence with the ablated GPX4-binding domain (*AAV-TM-M*) was also cloned into an *AAV8-TBG* vector. An *AAV8-TBG* vector containing a null cassette was used for the control group (*AAV-Con*). *AAV-TM, AAV-TM-M*, and *AAV-Con* were constructed by OBiO Technology Corp (Shanghai, China).

To generate hepatocyte-specific GPX4 knockdown mice (*AAV-GPX4 shRNA*), a scrambled sequence (targeting sequence: 5′-TTCTCCGAACGTGTCACGT-3′) (*AAV-Scr shRNA*) or an shRNA directed against mouse GPX4 shRNA (targeting sequence: 5′-TGGTCTGCCTGGATAAGT-3′) (*AAV-GPX4 shRNA*) were packaged into an AAV8-TBG vector. *AAV-Scr shRNA* and *AAV-GPX4 shRNA* were also obtained from OBiO Technology Corp. C57BL/6J background mice were administered the AAV8-TBG vectors (5 × 10^11^ viral genomes in 200 μL saline) via tail injection two weeks before the I/R procedure.

All mice were housed in a specific pathogen-free environment under controlled temperature (23 ± 2 °C) and light (12 h light/dark photocycle) conditions and were provided food and water *ad libitum*. Only 8- to 10-week-old male mice (24–27 g) were included in the study. For animal studies, grouping was performed based on animal genotype with no randomization or blinding used. No sample-size estimation was performed to ensure adequate power to detect a pre-specified effect size. Experiments involving animals were approved by the Ethics Committee of the Health Science Center from the School of Medicine at Yangtze University (approval no. YZLL2022-004) and were performed in accordance with the Guide for the Care and Use of Laboratory Animals.

### Hepatic I/R injury mouse model

Mice were anesthetized with sodium pentobarbital (50 mg/kg) and the liver was exposed by midline laparotomy. The hepatic artery branches of the portal vein responsive to left, lateral, and median lobes in the liver were partially occluded using an atraumatic microvascular clamp. Sham mice underwent the same operative procedure without vasculature occlusion. The clamp was released after 90 min of ischemia to initiate reperfusion for 1, 6, or 24 h. At the end of the procedure, liver and serum samples were immediately obtained for further analysis.

### Liver function analysis

Serum levels of ALT and AST were determined using a fully automated clinical chemistry analyzer (BS-800M; Mindray, Shenzhen, China) [9].

### H&E staining

Histopathological analysis was performed as previously described [9]. Paraffin-embedded liver sections (4 μm) were deparaffinized with xylene, rehydrated using an ethanol gradient, and stained with hematoxylin and eosin (H&E). Images were acquired using a light microscope (Olympus, Tokyo, Japan) and used to determine the size of the necrotic area and Suzuki scores after I/R injury. Necrotic areas in random fields on each slide were characterized by loss of architecture, vacuolization, karyolysis, and increased eosinophilia [55]. Histological criteria for the assessment of liver damage were assessed by Suzuki score (congestion, vacuolization, and necrosis) as previously described [56].

### Immunofluorescence staining

Immunofluorescence staining was performed as previously described [9, 57]. After deparaffinizing and rehydrating the liver sections, antigen retrieval was performed. Next, the sections were incubated with 10% bovine serum albumin (BSA) for 1 h at 37 °C, and then incubated with primary antibodies overnight at 4 °C. The primary antibodies included: TMEM16A (Cat No. ab53212; 1:100, Abcam, Cambridge, MA), HNF4 (Cat No. ab41898; 1:100, Abcam), CD68 (Cat No. MCA1957; 1:100, Bio-Rad, Hercules, CA, USA), LY6G (Cat No. 551459; 1:50, BD Biosciences, San Jose, CA, USA), and CD3 (Cat No. ab16669; 1:100, Abcam). Subsequently, the sections were incubated with fluorescence-labeled secondary antibodies for 1 h and further stained with DAPI (Invitrogen, Carlsbad, CA, USA) for 10 min at room temperature in the dark.

TUNEL staining (Millipore, Billerica, MA, USA) was performed to evaluate cell death in liver tissue slides, according to manufacturer instructions. The slides were viewed under a laser scanning confocal microscope (LSM800; Zeiss, Jena, Germany).

### Immunohistochemistry

Immunohistochemistry was performed as previously described [9, 58]. The sections were treated with 3% H_2_O_2_ for 10 min to quench endogenous peroxidase activity and then blocked with 10% BSA for 1 h at 37 °C. The sections were incubated with 4-HNE primary antibody (Cat No. ab48506; 1:100, Abcam) overnight at 4 °C, followed by incubation with biotinylated secondary antibodies (Cat No. AP180B; 1:200, Millipore) for 1 h at 37 °C. Subsequently, the sections were incubated with horseradish peroxidase (HRP)–labeled avidin (Cat No. SA-5004; 1:100, Vector Laboratories, Burlingame, CA, USA) for 30 min. Finally, the sections were visualized by staining with 3,3-diaminobenzidine (Zhongshan Golden Bridge Biological Technology Co., Ltd., Beijing, China) and counterstained with hematoxylin. The sections were viewed under a light microscope (Olympus).

### Isolation of primary hepatocytes and non-parenchymal cells

Primary hepatocytes were isolated from mice as described previously [9]. Briefly, after being anesthetized with sodium pentobarbital, mice were perfused through the portal vein using Hanks’ Balanced Salt Solution without Ca^2+^ and Mg^2+^. Afterward, the liver was perfused with 0.05% collagenase type IV (Thermo Fisher Scientific, Waltham, MA, USA). The liver was excised and filtered through a 70 μm cell filter (BD Biosciences). Dissociated hepatocytes and non-parenchymal cells (NPCs) were separated using discontinuous Percoll (GE Healthcare, Piscataway, NJ, USA) gradient centrifugation. The cells were collected in DMEM (Gibco, Thermo Fisher Scientific) supplemented with 10% fetal bovine serum (FBS) (Gibco) and 1% penicillin-streptomycin (Gibco) and cultured in an incubator at 37 °C under 5% CO_2_.

### Cell lines and culture

HEK293T cells and L02 cells were purchased from the Type Culture Collection of the Chinese Academy of Sciences (Shanghai, China). All cell lines used in this study were cultured in DMEM containing 10% FBS and 1% penicillin-streptomycin in a humidified incubator maintained at 37 °C and 5% CO_2_. All cells were authenticated by STR profiling and tested for mycoplasma contamination.

### Hypoxia/reoxygenation (H/R) challenge

Hepatocytes were challenged with hypoxia in DMEM without serum or glucose (Gibco) under 1% oxygen condition (hypoxia) for 4 h and then incubated under normoxic conditions with DMEM containing 10% FBS for the indicated time points.

### Current recordings in hepatocytes

Patch–clamp recordings were performed at room temperature on isolated primary hepatocytes using an Axopatch 200B patch–clamp amplifier (Axon Instruments, Foster City, CA, USA) as previously described [9]. Patch pipettes were pulled from borosilicate capillary glass with a Sutter P-97 horizontal puller (Sutter Instrument Co., Novato, CA, USA). When filled with pipette solution, the resistance of the pipettes was 3–6 MΩ. The currents were sampled at 5 kHz and filtered at 2 kHz before analysis using pCLAMP8.0 software (Axon Instruments). Subsequently, the current was measured using 500 ms voltage steps from −100 mV to +100 mV in +20 mV increments at 5 s intervals. The extracellular solution contained 125 mmol/L N-methyl-d-glucamine (NMDG)-Cl, 5 mmol/L KCl, 1.5 mmol/L CaCl_2_, 1 mmol/L MgSO_4_, 10 mmol/L HEPES, and 10 mmol/L glucose, and the pH was adjusted to 7.4 with NMDG. The pipette solution contained 130 mmol/L CsCl, 1 mmol/L Mg·ATP, 1.2 mmol/L MgCl_2_, 10 mmol/L HEPES, 2 mmol/L EGTA, and 1.639 mmol/L CaCl_2_, and the pH was adjusted to 7.4 with CsOH. The intracellular Ca^2+^ concentration was 500 nmol/L.

### Flow cytometry analysis of non-parenchymal cells

The NPCs isolated from sham- or I/R-treated mice were incubated with anti-CD16/32 antibody (Cat No. 553141; BD Pharmingen, Franklin Lakes, NJ, USA) to interrupt non-specific binding of NPCs to the Fcγ receptor. Subsequently, NPCs were stained with APC-labeled anti-F4/80 antibody (Cat No. clone BM8, Biolegend San Diego, CA, USA), PE-conjugated anti-LY6G antibody (Cat No. 12-9668-82, eBioscience, San Diego, CA, USA), and PerCP/Cy5.5-conjugated anti-CD11b antibody (Cat No. 45-0112-80; eBioscience). *CD11d*^*+*^*LY6G*^*+*^ and *CD11b*^*+*^*F4/80*^*+*^ cells were identified as neutrophils and Kupffer cells, respectively. Flow cytometry was performed using the BD FACSVerse Flow Cytometer (BD Biosciences) and data were analyzed using FlowJo software (Tree Star, Ashland, OR, USA).

### Cytokines and chemokines assays

Inflammatory cytokines (TNF-α, IL-1β, and IL-6) and chemokines (C-X-C motif chemokine ligand 1 [CXCL-1] and monocyte chemoattractant protein-1 [MCP-1]) in serum and primary hepatocytes were measured by commercially available enzyme-linked immunosorbent assay kits (R&D Systems, Minneapolis, MN, USA).

### Cell counting kit-8 (CCK-8) and lactate dehydrogenase release assay

Cell viability was measured by CCK-8 (Dojindo, Kumamoto, Japan) and necrotic cells were assessed by the release of lactate dehydrogenase (LDH) in the medium using the LDH Cytotoxicity Assay Kit (Promega, Madison, WI, USA). The kits were used according to manufacturer instructions.

### Quantitative real-time PCR and western blot analysis

Total RNA was isolated from hepatocytes or liver tissues using TriPure Isolation Reagent (Roche, Basel, Switzerland) according to manufacturer instructions. RNA (2 μg) was reverse transcribed into cDNA using the Transcriptor First Strand cDNA Synthesis Kit (Roche) and real-time PCR was performed using the SYBR Green PCR Master Mix (Bio-Rad Laboratories) on a MyiQ Single Color Real-time PCR Detection System (Bio-Rad Laboratories). mRNA expression was normalized to that of β-actin. The primer sequences used in the study are listed in Supporting Table S3.

Western blotting was performed as previously described [9, 59]. Briefly, total proteins were extracted from tissues and cell samples using radioimmunoprecipitation assay lysis buffer supplemented with a protease inhibitor cocktail (Roche) and phosphatase inhibitor (Roche). The protein concentration was quantified using the Pierce BCA Protein Assay Kit (Thermo Fisher Scientific). The sample was centrifuged at 12,000×*g* for 12 min, resuspended in SDS loading buffer, and boiled at 95 °C for 10 min. Proteins were separated by SDS-PAGE and transferred to polyvinylidene fluoride membranes (Millipore). The membranes were blocked with 5% nonfat milk and incubated with primary antibodies overnight at 4 °C. Subsequently, the membranes were incubated with HRP-conjugated secondary antibodies for 1 h at room temperature and developed with ECL reagent (Thermo Fisher Scientific). The images were captured by the ChemiDoc MP Imaging System (Bio-Rad). β-actin served as the internal control. The antibodies used in western blotting are listed in Supporting Table S4.

### Plasmid construction and transfection

Human full-length TMEM16A and truncated TMEM16A (1–319, 320–541, 542–732, and 733–986 aa), in conjunction with TM-M (755–782aa deleted from full-length TMEM16A gene) amplified from the human cDNA library, were cloned into pEGFP-N1 vectors and tagged with Flag. Full-length human GPX4 was cloned into pcDNA3.1(+)-mCherry tagged with hemagglutinin (HA). Ubiquitin (Ub) was cloned into a pcDNA3.1(+) vector tagged with Myc. All primers used for plasmid construction are listed in Supplementary Table S5. Plasmid transfection was performed using Lipofectamine 2000 reagent (Invitrogen) according to manufacturer instructions.

### Immunoprecipitation (IP) assay

HEK293T cells or L02 cells were analyzed via IP assay, as previously described [9, 57]. After plasmid transfection, cells were lysed in an IP buffer on ice. A small aliquot of the lysate was used as input, and the remaining lysate was incubated with a mixture of Protein A Magnetic Beads (Bio-Rad) and Protein G Magnetic Beads (Bio-Rad) together with corresponding antibodies for at least 4 h at 4 °C with rotation. Normal rabbit IgG (Cat No. 2729; Cell Signaling Technology, Danvers, MA, USA) was used as the negative control. After washing the beads four times with IP buffer, the immunoprecipitates bound to the beads were collected and analyzed by western blotting.

### Transmission electron microscopy

TEM was performed as previously described [58]. Primary hepatocytes were fixed with 0.1 M sodium cacodylate buffer containing 2.5% glutaraldehyde for 4 h, treated with 2% OsO4 in 0.1 M sodium cacodylate buffer for 2 h, and finally incubated overnight in 1% aqueous uranyl acetate. After dehydration using an ethanol gradient, the specimens were embedded in epoxy resin. Polymerization was carried out at 80 °C for 24 h. The collected ultra-thin sections from copper grids were stained with uranyl acetate and lead citrate. The ultrastructure of the hepatocytes was examined using a JEM2000EX transmission electron microscope (JEOL, Tokyo, Japan) and mitochondrial lengths were quantified.

### Detection of lipid peroxides

After H/R treatment, the primary hepatocytes were washed twice with PBS and incubated with DMEM containing 2 μmol/L BODIPY 581/591 C11 (Invitrogen) for 30 min. After washing with PBS, the hepatocytes were fixed with 4% paraformaldehyde and stained with DAPI for 10 min. Images of green fluorescence (490–530 nm) and red fluorescence (570–610 nm) emissions were captured upon excitation at 488 and 568 nm, respectively, using a laser scanning confocal microscope (LSM800; Zeiss). The BODIPY 581/591 C11 value was calculated as the ratio of the green fluorescence (indicating the oxidized form of BODIPY 581/591 C11) to red fluorescence (indicating the non-oxidized form of BODIPY 581/591 C11).

### Iron, glutathione (GSH), malondialdehyde (MDA), and NADPH assays

The relative iron concentration in serum was assessed using the Iron Assay Kit (Abcam). The hepatic MDA concentration was measured using the Lipid Peroxidation (MDA) Assay Kit (Abcam). The relative GSH concentration in liver tissue lysates was detected using a Glutathione Assay Kit (Sigma-Aldrich). The relative NADPH concentration in liver sections was assessed using the NADPH Assay Kit (Abcam). All kits and reagents were used according to manufacturer instructions.

### Statistical analysis

IBM SPSS version 19.0 (IBM Corp., Armonk, NY, USA) was used to conduct statistical analyses. All inclusion/exclusion criteria were pre-established, and no animals or samples were excluded from the analysis. All data were expressed as the mean ± standard deviation. For comparisons between two groups, normally distributed data were analyzed using the two-tailed Student *t*-test, whereas skewed data were analyzed using the Mann–Whitney *U* test. To compare more than two groups, data with homogeneity of variance were compared using one-way analysis of variance (ANOVA) followed by the Bonferroni post hoc test, whereas data with heteroscedastic variance were analyzed using Tamhane’s T2 method. Two-way ANOVA was performed for multiple comparisons involving two independent variables followed by the Bonferroni post hoc test. A *P*-value < 0.05 was considered statistically significant. The variance is similar between groups that are being statistically compared.

## Supplementary information

Supplementary materials

Original western blots

Reproducibility checklist

## Data Availability

The data of this study can be obtained from the corresponding author upon reasonable request.
