# Supplementary material for: Hepatocyte-specific TMEM16A deficiency alleviates hepatic ischemia/reperfusion injury via suppressing GPX4-mediated ferroptosis
Source: Cell Death Dis. 2022 Dec 26;13(12):1072. doi: 10.1038/s41419-022-05518-w (PMC9792590; doi:10.1038/s41419-022-05518-w)
Supplement: Supplementary file 1 — Supplementary materials [file 41419_2022_5518_MOESM1_ESM.docx]

**Hepatocyte-specific TMEM16A deficiency alleviates hepatic ischemia/reperfusion injury via suppressing GPX4-mediated ferroptosis**

Jiawei Guo*^1^, Zihao Song*^2^, Jie Yu*^3^, Chengyi Li^2^, Chenchen Jin^4^, Wei Duan^5^, Xiu Liu^6^, Yingying Liu^7^, Shuai Huang^8^, Yonghua Tuo^9^, Fei Pei^10,11^, Zhengyang Jian^12^, Pengyu Zhou^6^, Shaoyi Zheng^6^, Zhaowei Zou^3^, Feng Zhang^13^, Quan Gong^2^, Sijia Liang^14^

^1^Department of Pharmacology, School of Medicine, Yangtze University, Jingzhou, China; ^2^Department of Immunology, School of Medicine, Yangtze University, Jingzhou, China; ^3^Department of General Surgery, Zhujiang Hospital, Southern Medical University, Guangzhou, China; ^4^Center for Neuro-Metabolism and Regeneration Research, The Bioland Laboratory, Guangzhou, China; ^5^Department of Oncology, Jingzhou Hospital Affiliated to Yangtze University, Jingzhou China; ^6^Department of Cardiovascular Surgery, Nanfang Hospital, Southern Medical University, Guangzhou, China; ^7^Guangzhou Women and Children's Medical Center, Guangdong Provincial Clinical Research Center for Child Health, Guangzhou, China; ^8^Department of Orthopaedic Surgery, The Second Affiliated Hospital of Guangzhou Medical University, Guangzhou, China; ^9^Department of Neurosurgery, The Second Affiliated Hospital of Guangzhou Medical University, Guangzhou, China; ^10^Department of Critical Care Medicine, The First Affiliated Hospital, Sun Yat-Sen University, Guangzhou, China; ^11^Guangdong Clinical Research Center for Critical Care Medicine, Guangzhou, China; ^12^Center For Drug Inspection of Guizhou Medical Products Administration, Guiyang, China; ^13^Department of Radiology, University of Washington School of Medicine, Seattle, WA, USA; ^14^Department of Pharmacology, Zhongshan School of Medicine, Sun Yat-Sen University, Guangzhou, China.

**Running title:** TMEM16A, ferroptosis, and hepatic I/R

**Corresponding authors:**

Jaiwei Guo, Ph.D. (ORCID: 0000-0001-9471-2508)

Department of Pharmacology, School of Medicine, Yangtze University

1 Nanhuan Road, Jingzhou 434023, China

Email: [guojw9@mail2.sysu.edu.cn](mailto:guojw9@mail2.sysu.edu.cn)

Tel: +86-0716-8060609

Quan Gong, Ph.D. (ORCID: 0000-0003-4094-7883)

Department of Immunology, School of Medicine, Yangtze University

1 Nanhuan Road, Jingzhou 434023, China

Email: [gongquan@yangtzeu.edu.cn](mailto:gongquan@yangtzeu.edu.cn)

Tel: +86-0716-8060609

Sijia Liang, Ph.D. (ORCID: 0000-0002-4014-6325)

Department of Pharmacology, Zhongshan School of Medicine, Sun Yat-Sen University

74 Zhongshan 2 Rd, Guangzhou, 510080, China.

E-mail: [liangsj5@mail.sysu.edu.cn](mailto:liangsj5@mail.sysu.edu.cn)

Tel: +86-020-87331155

**Supporting Tables**

**Table S1. Patient characteristics**

| Patient Number | Gender | Age  (years) | Etiology of disease | ALT  (U/L) | ALT  (U/L) |
| --- | --- | --- | --- | --- | --- |
| 1 | Male | 39 | Hepatic cyst | 940 | 1263 |
| 2 | Male | 46 | Hepatic cyst | 243 | 606 |
| 3 | Female | 52 | Hepatic cyst | 1114 | 1756 |
| 4 | Male | 25 | Hepatic cyst | 1287 | 1877 |
| 5 | Male | 57 | Hepatic cyst | 1647 | 2187 |
| 6 | Female | 46 | Hepatic cyst | 244 | 963 |
| 7 | Male | 45 | Hepatic cyst | 1454 | 2054 |
| 8 | Female | 32 | Hepatic cyst | 1825 | 2559 |
| 9 | Male | 48 | Hepatic hemangioma | 119 | 284 |
| 10 | Female | 54 | Hepatic hemangioma | 1202 | 1844 |
| 11 | Male | 40 | Hepatic hemangioma | 2724 | 3008 |
| 12 | Female | 47 | Hepatic hemangioma | 1823 | 2516 |
| 13 | Female | 36 | Hepatic hemangioma | 446 | 1109 |
| 14 | Female | 43 | Hepatic hemangioma | 1924 | 2730 |
| 15 | Male | 36 | Hepatic hemangioma | 1401 | 1917 |
| 16 | Male | 39 | Hepatic hemangioma | 2595 | 2946 |
| 17 | Female | 54 | Hepatic hemangioma | 98 | 243 |
| 18 | Female | 48 | Hepatic hemangioma | 1754 | 2367 |
| 19 | Male | 47 | Hepatic hemangioma | 799 | 1209 |
| 20 | Female | 52 | Hepatic hemangioma | 1444 | 1998 |
| 21 | Male | 40 | Intrahepatic cholelithiasis | 210 | 538 |
| 22 | Female | 43 | Intrahepatic cholelithiasis | 2025 | 2779 |
| 23 | Male | 52 | Intrahepatic cholelithiasis | 1004 | 1670 |
| 24 | Female | 44 | Intrahepatic cholelithiasis | 943 | 1283 |
| 25 | Female | 46 | Intrahepatic cholelithiasis | 1752 | 2229 |
| 26 | Female | 56 | Intrahepatic cholelithiasis | 2503 | 2941 |
| 27 | Male | 47 | focal nodular hyperplasia | 241 | 603 |
| 28 | Female | 32 | focal nodular hyperplasia | 1042 | 1720 |
| 29 | Male | 56 | PECOMA | 620 | 1150 |
| 30 | Female | 41 | PECOMA | 968 | 1394 |

ALT, aspartate amino transferase; AST, alanine amino transferase; PECOMA, perivascular epithelioid cell tumor.

**Table S2. Primers for genotyping**

| Gene | Primer (5′-3′) |
| --- | --- |
| TMEM16A F1 | GGTATCACCCAAGGTAACCATCCA |
| TMEM16A R1 | CAACCCTCTCTATCCCTGTCACATG |
| TMEM16A F2 | TGATTCTGATAGCAAATGAGGCAGAT |
| TMEM16A R2 | AGGTTATCATAGCTCAGTTCACAAGCTTT |
| Alb Cre F | GAAGCAGAAGCTTAGGAAGATGG |
| Alb Cre R | TTGGCCCCTTACCATAACTG |

**Table S3. Primers for real-time PCR analysis**

| Gene | Forward primer (5′-3′) | Reverse primer (5′-3′) |
| --- | --- | --- |
| TMEM16A  (human) | CTGATGCCGAGTGCAAGTATG | AGGGCCTCTTGTGATGGTACA |
| TMEM16A  (mouse) | CCGTGCCAGTCACCTTTTTG | GAAGCCTGTGAGGTCCCATC |
| β-actin  (human) | GCTTCTCCTTAATGTCACGC | CCCACACTGTGCCCATCTAC |
| β-actin  (mouse) | TATTGGCAACGAGCGGTTCC | GGCATAGAGGTCTTTACGGATGTC |
| TNFα  (mouse) | AGCCGATGGGTTGTACCTTGTCTA | TGAGATAGCAAATCGGCTGACGGT |
| IL-1β  (mouse) | CCGTGGACCTTCCAGGATGA | GGGAACGTCACACACCAGCA |
| MCP1  (mouse) | TGGCTCAGCCAGATGCAGT | CCAGCCTACTCATTGGGATCA |
| Cxcl-1  (mouse) | TGCACCCAAACCGAAGTCATAGCC | AGCCAGCGTTCACCAGACAGGT |
| GPX4  (mouse) | CCGGCTACAACGTCAAGTTT | CACGCAGCCGTTCTTATCA |
| Ptgs2  (mouse) | GGGAGTCTGGAACATTGTGAA | CCCCAAAGGGATGAGAGTTC |

**Table S4. Antibodies for western blot analysis**

| Antibodies | Source | Cat NO. | Dilution |
| --- | --- | --- | --- |
| β-actin | Proteintech | 20536-1-AP | 1:2000 |
| GAPDH | Proteintech | 60004-1-Ig | 1:2000 |
| Flag | Proteintech | 20543-1-AP | 1:2000 |
| HA | Santa Cruz Biotechnology | sc-7392 | 1:500 |
| Myc | Santa Cruz Biotechnology | sc-40 | 1:500 |
| p-P65 | Cell Signaling Technology | 3033 | 1:1000 |
| P65 | Cell Signaling Technology | 4764 | 1:1000 |
| p-IKKβ | Abcam | ab59195 | 1:1000 |
| IKKβ | Cell Signaling Technology | 2370 | 1:1000 |
| p-IKBα | Cell Signaling Technology | 9246 | 1:1000 |
| IKBα | Cell Signaling Technology | 4814 | 1:1000 |
| Cleaved Caspase3 | Cell Signaling Technology | 9664 | 1:1000 |
| Caspase3 | Cell Signaling Technology | 9662 | 1:1000 |
| p-MLKL | Abcam | ab196436 | 1:1000 |
| MLKL | Millipore Sigma | MABC604 | 1:1000 |
| NLRP3 | Abcam | ab214185 | 1:1000 |
| Cleaved Caspase1 | Cell Signaling Technology | 89332 | 1:1000 |
| Caspase1 | Cell Signaling Technology | 24232 | 1:1000 |
| GPX4 | Abcam | ab125066 | 1:2000 |
| TMEM16A | Abcam | Ab53212 | 1:1000 |
| Ubiquitin | Santa Cruz Biotechnology | sc-8017 | 1:500 |

**Table S5. Primers for plasmid construction**

| Gene | Forward primer (5′-3′) | Reverse primer (5′-3′) |
| --- | --- | --- |
| Flag-TMEM16A | CGCAAATGGGCGGTAGGCGTG | GACACGCTGAACTTGTGGC |
| Flag-TMEM16A  (1-320 aa) | CGCAAATGGGCGGTAGGCGTG | TGGCATCGCCCTCGCCCTC |
| Flag-TMEM16A  (321-542 aa) | CGCAAATGGGCGGTAGGCGTG | GAAATTTGTGATGCTATTGC |
| Flag-TMEM16A  (543-732 aa) | CGCAAATGGGCGGTAGGCGTG | GAAATTTGTGATGCTATTGC |
| Flag-TMEM16A  (733-986 aa) | CGCAAATGGGCGGTAGGCGTG | GAAATTTGTGATGCTATTGC |
| Flag-TMEM16A-M | CGCAAATGGGCGGTAGGCGTG | CCTCACATTGCCAAAAGACG |
| HA-GPX4 | CGCAAATGGGCGGTAGGCGTG | GATCTCGAACTCGTGGCCGT |
| Myc-  ubiquitin | CGCAAATGGGCGGTAGGCGTG | TTATTAGGAAAGGACAGTGGG |

**Supporting Figures**

**
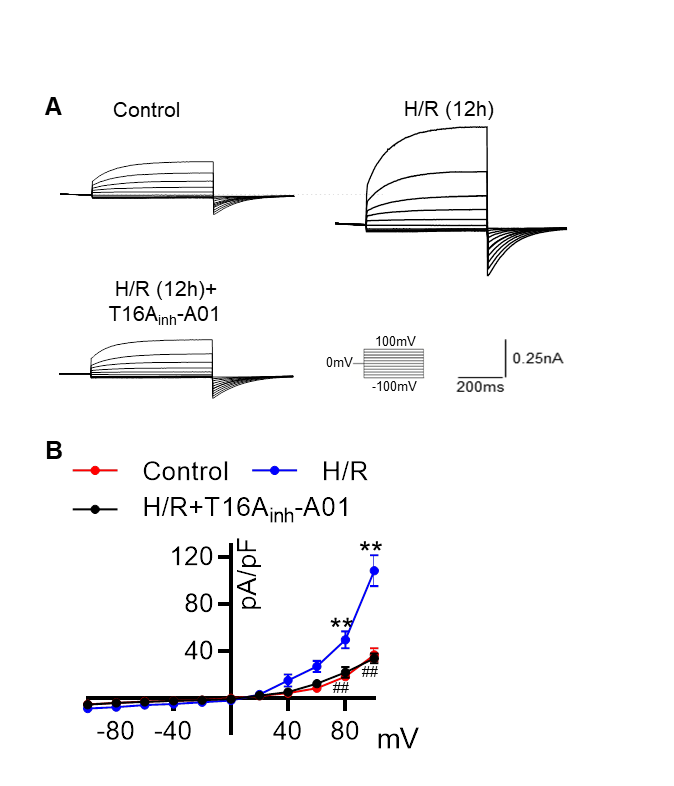
**

**Figure S1. TMEM16A currents in primary hepatocytes are activated by H/R insult.** (A) Whole-cell TMEM16A currents in primary hepatocytes after 4 h of hypoxia and 12 h of reoxygenation (H/R) in the presence of 500 nM intracellular Ca^2+^ ([Ca^2+^]_i_) were recorded in responding to voltage ramps from −100 to +100 mV. The reversal potential at 0.35 ± 2.72 mV was near the equilibrium potential for Cl^−^ (0 mV) under the recording conditions. The activated currents were blocked after treatment with T16Ainh-A01 (10 µM), an inhibitor of TMEM16A. (B) Corresponding current/voltage (I/V) plot of the recorded currents (n = 4). **P < 0.01 versus control; ##P < 0.01 versus H/R. Data were presented as the mean ± SD.

**
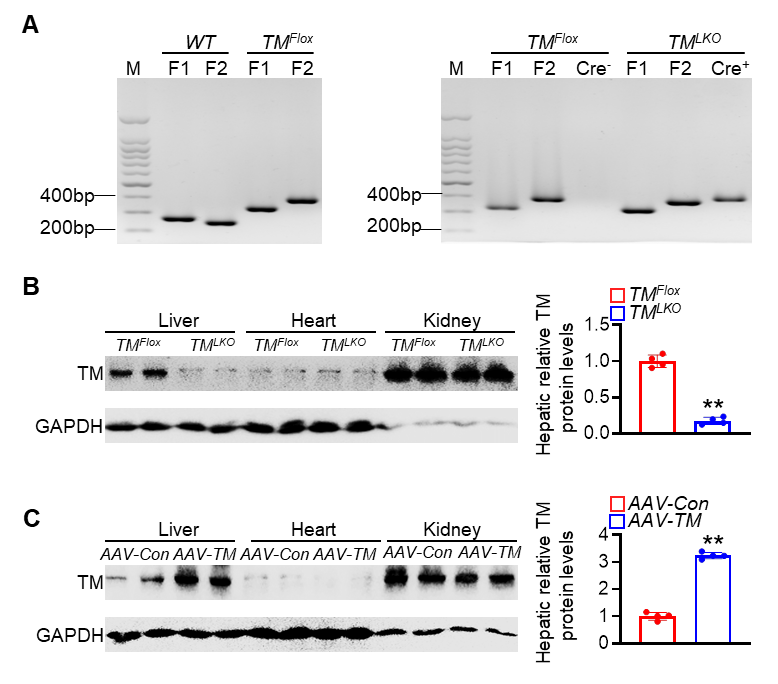
**

**Figure S2. Verification of TMEM16A knockout and overexpression in liver.** (A) Representative images of PCR amplification products analyzed by SDS-PAGE. *TM^LKO^* mice were considered to include F1 (325 bp), F2 (379 bp), and Cre (390 bp). *TM^Flox^* mice contained F1 and F2 but did not contain Cre. The bands at 264 and 239 bp represent the wild-type allele. Lane M, 100 bp DNA ladder. (B) TMEM16A protein expression in the liver, heart, and kidney of *TM^Flox^* and *TM^LKO^* mice (n = 4). **P < 0.01 versus *TM^Flox^*. (C) TMEM16A expression in the liver, heart, and kidney of *AAV-Con*- and *AAV-TM*-treated mice (n = 4). **P < 0.01 versus *AAV-Con*. Data were presented as the mean ± SD.


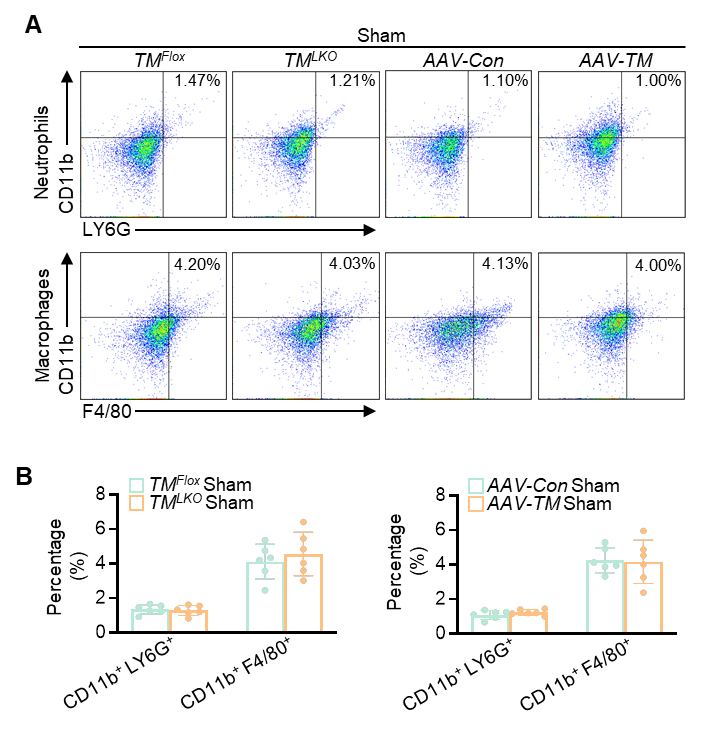


**Figure S3. Effects of TMEM16A on neutrophil and macrophage infiltration in liver tissue under sham operation.** (A) Representative flow cytometry histogram showing neutrophils and macrophages in liver samples from the indicated groups after sham operation. (B) Quantification of neutrophils and macrophages as measured by flow cytometry. No significant difference in inflammatory cell infiltration was observed in mice that received sham treatment (n = 6). Data were presented as the mean ± SD.


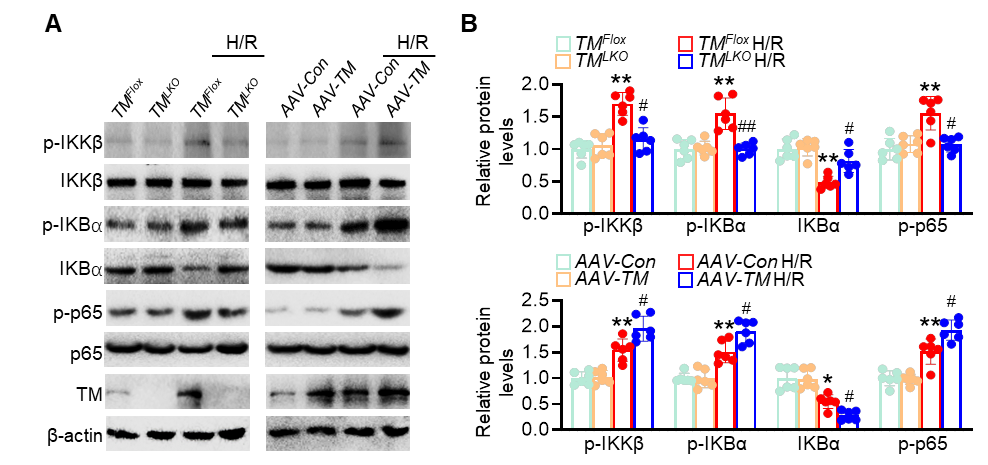


**Figure S4. TMEM16A enhances H/R-induced inflammation in hepatocytes.** (A) Representative western blot of total and phosphorylated IKKβ, IκBα, and p65 levels in hepatocytes isolated from *TM^LKO^* or *AAV-TM*-treated mice and their counterparts after H/R treatment (4 h of hypoxia and 12 h of reoxygenation). (B) Densitometric analysis of the expression of the above proteins (n = 6). *P < 0.05, **P < 0.01 versus *TM^Flox^* or *AAV-Con*; #P < 0.05, ##P < 0.01 versus *TM^Flox^* H/R or *AAV-Con* H/R. Data were presented as the mean ± SD.


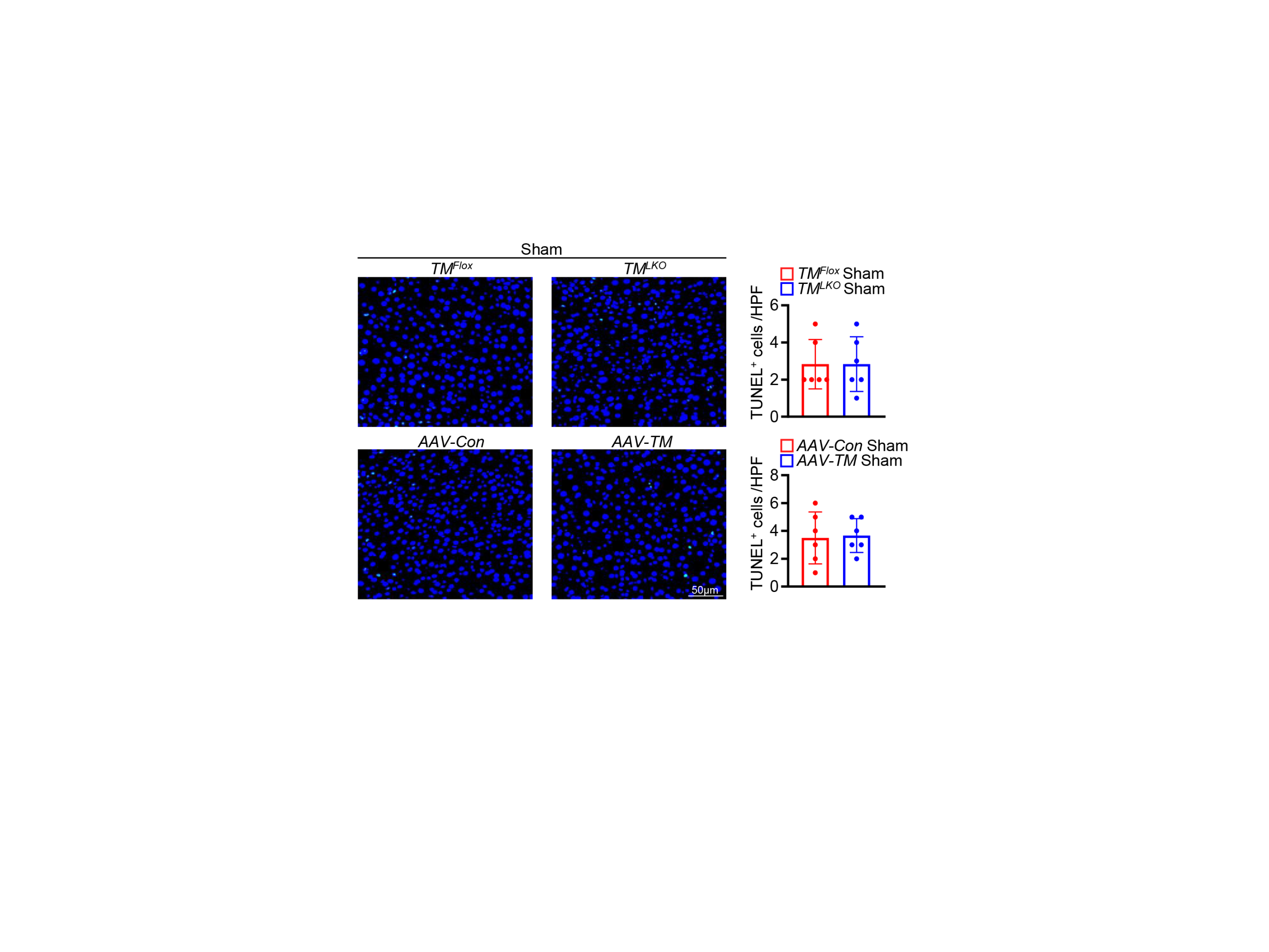


**Figure S5. Effects of TMEM16A on cell death in liver tissue under sham operation.** Representative images and quantification of TUNEL immunofluorescence analyses in liver sections from *TM^Flox^*, *TM^LKO^*, *AAV-Con*-treated*,* and *AAV-TM*-treated mice under sham operation (n = 6). Green fluorescence was identified as TUNEL-positive cells and blue fluorescence exhibited DAPI-positive cells. Data were presented as the mean ± SD.


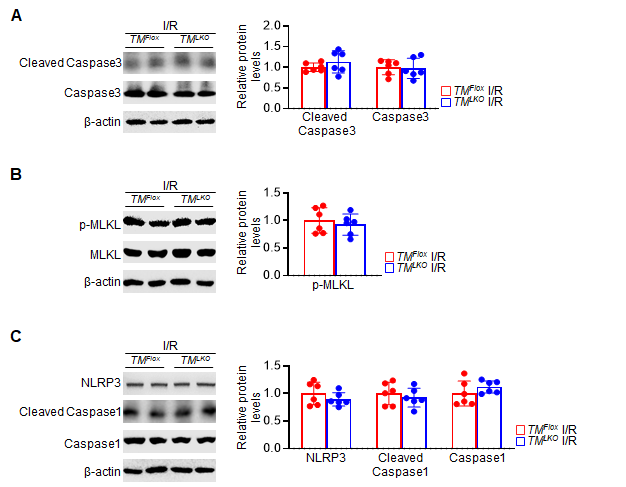


**Figure S6. Effects of TMEM16A ablation on markers of apoptosis, necroptosis, and pyroptosis.** (A-C) Western blot analysis of hepatic protein levels of (A) cleaved caspase-3, caspase-3, (B) phosphorylated MLKL, (C) NLRP3, cleaved caspase-1, and caspase-1 in *TM^LKO^* mice and their counterparts after 90 min of ischemia followed by 24 h of reperfusion (n = 6). Data were presented as the mean ± SD.


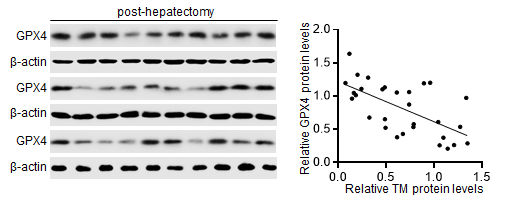


**Figure S7. Correlation between TMEM16A and GPX4 expression in human liver samples.** Western blot analysis of GPX4 protein expression in human liver samples obtained post-hepatectomy (n = 30). Pearson correlation analysis between post-hepatectomy hepatic TMEM16A protein expression and GPX4 protein expression (r^2^ = 0.3954, P < 0.01).

**
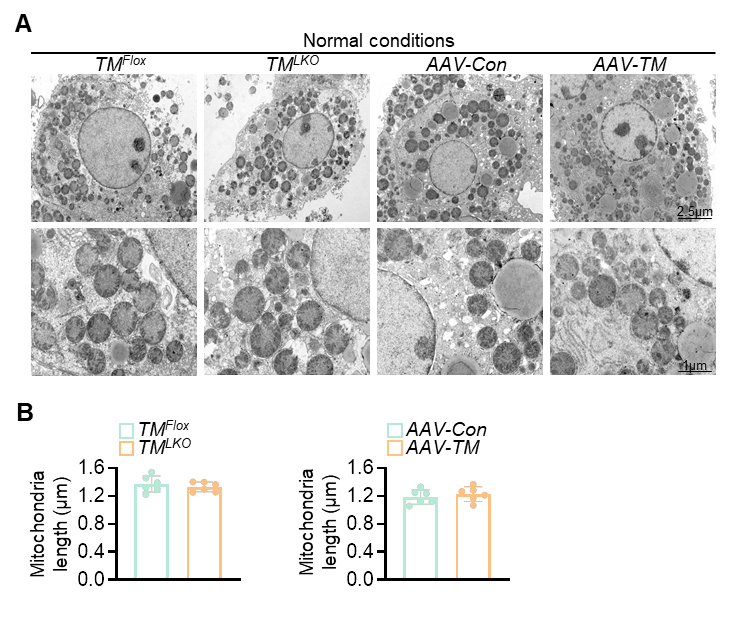
**

**Figure S8. Role of TMEM16A in hepatocyte ferroptosis under normal conditions.** (A) Representative TEM images of the ultrastructure of mitochondria in hepatocytes isolated from indicated mice under normal conditions. (B) Quantification of mitochondrial length. TMEM16A knockout or overexpression had no effect on the mitochondrial morphology of hepatocytes under basal conditions (n = 6). Data were presented as the mean ± SD.


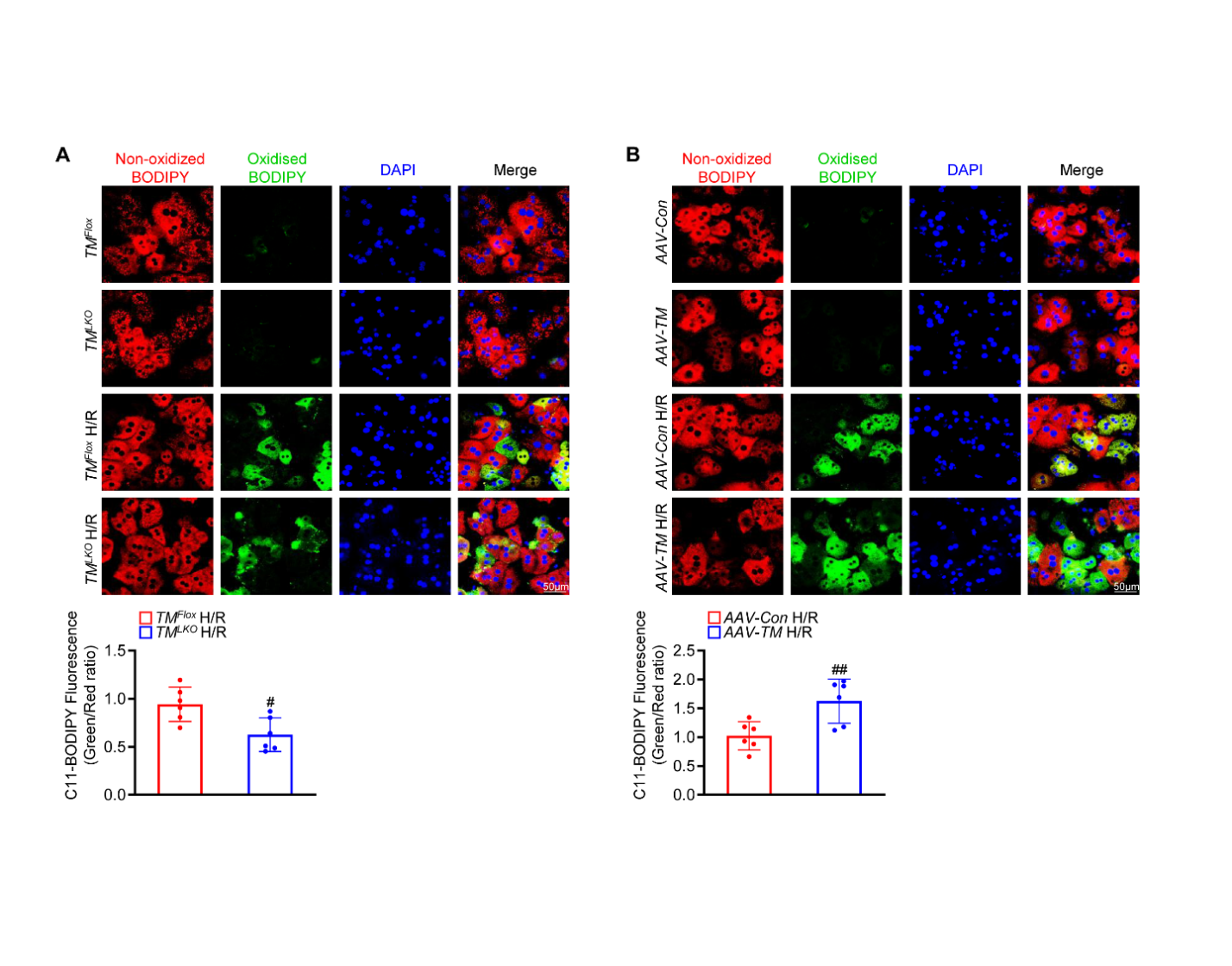


**Figure S9. Hepatocyte-specific TMEM16A regulates lipid ROS production in hepatocytes.** (A, B) Representative images of C11-BODIPY staining in primary hepatocytes isolated from (A) *TM^LKO^* and (B) *AAV-TM*-treated mice and their counterparts treated with 4 h of hypoxia and 12 h of reoxygenation (n = 6). Red, reduced form of C11-BODIPY; green, oxidized form of C11-BODIPY. #P < 0.05 versus *TM^Flox^* H/R; ##P < 0.01 versus *AAV-Con* H/R. Data were presented as the mean ± SD.

**
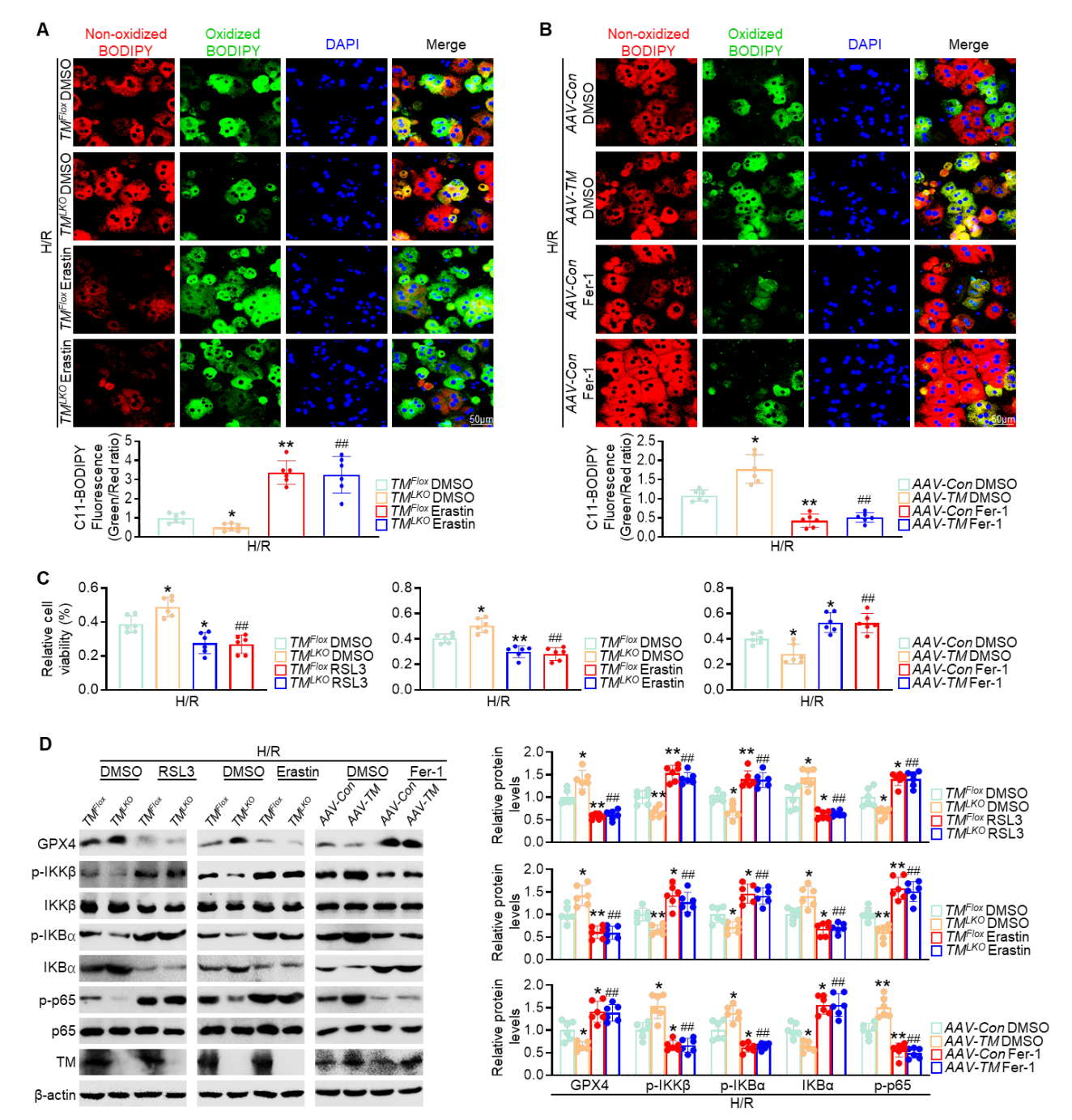
**

**Figure S10. Ferroptosis is involved in TMEM16A-mediated hepatic I/R injury.** (A, B) Representative images and quantitation of C11-BODIPY staining of primary hepatocytes isolated from indicated mice treated with (A) Erastin (10 μmol/L) or (B) Ferrostatin-1 (Fer-1, 2 μmol/L) for 8 h followed by 4 h of hypoxia and 12 h of reoxygenation (n = 6). Red, reduced form of C11-BODIPY; green, oxidized form of C11-BODIPY. *P < 0.05, **P < 0.01 versus *TM^Flox^* DMSO or *AAV-Con* DMSO; ##P < 0.01 versus *TM^LKO^* DMSO or *AAV-TM* DMSO. (C) Cell viability after the aforementioned treatment. *P < 0.05, **P < 0.01 versus *TM^Flox^* DMSO or *AAV-Con* DMSO; ##P < 0.01 versus *TM^LKO^* DMSO or *AAV-TM* DMSO. (D) Expression of proteins involved in NF-κB signaling in hepatocytes after the aforementioned treatment (n = 6). *P < 0.05, **P < 0.01 versus *TM^Flox^* DMSO or *AAV-Con* DMSO; ##P < 0.01 versus *TM^LKO^* DMSO or *AAV-TM* DMSO. Data were presented as the mean ± SD.


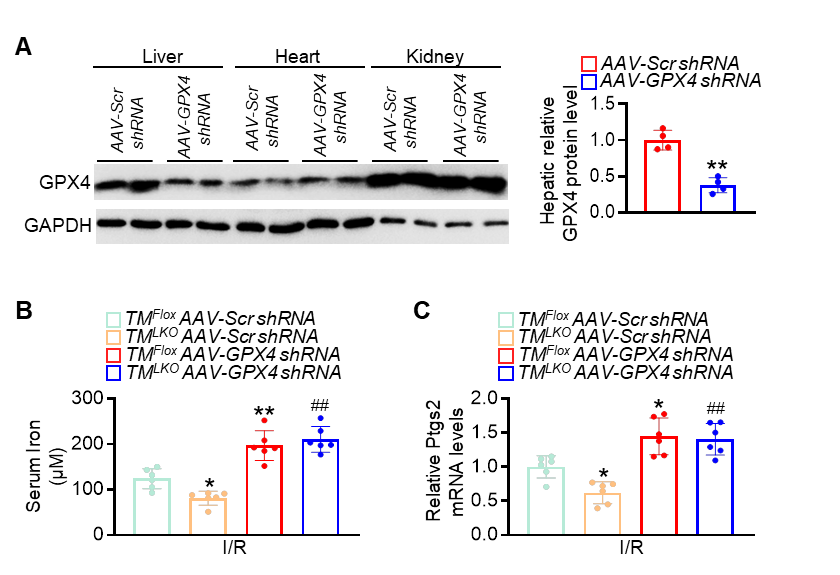


**Figure S11. Effects of GPX4 knockdown on TMEM16A-mediated ferroptosis.** (A) GPX4 expression in the liver, heart, and kidney of *AAV-Scr shRNA-* or *AAV-GPX4 shRNA*-treated mice (n = 4). **P < 0.01 versus *AAV-Scr shRNA***.** (B, C) Serum iron levels and hepatic PTGS2 expression in *TM^LKO^* and *TM^Flox^* mice treated with *AAV-GPX4 shRNA* prior to 90 min of hepatic ischemia followed by 24 h of reperfusion (n = 6). *P < 0.05, **P < 0.01 versus *TM^Flox^ AAV-Scr shRNA*; ##P < 0.01 versus *TM^LKO^* *AAV-Scr shRNA*. Data were presented as the mean ± SD.


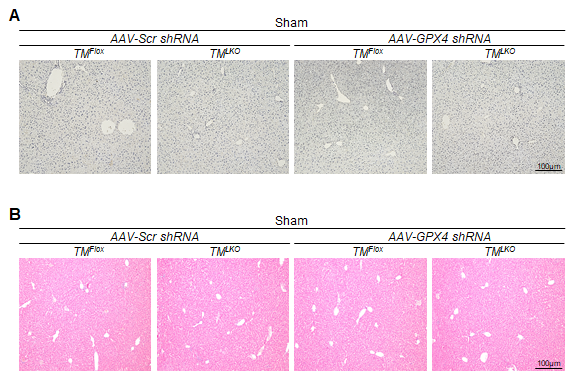


**Figure S12. Effects of GPX4 knockdown on TMEM16A-mediated ferroptosis and liver injury under sham operation.** (A) Representative images showing 4-HNE immunohistochemical staining from *TM^Flox^* and *TM^LKO^* mice injected with *AAV-GPX4 shRNA* via tail vein two weeks prior to sham performance (n = 6). (B) Representative H&E staining of liver sections in indicated mice (n = 6).

\

**
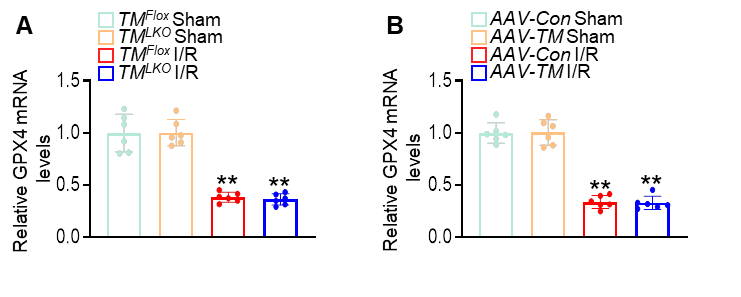
**

**Figure S13. Effects of TMEM16A on GPX4 mRNA levels.** Hepatic GPX4 mRNA levels in (A) *TM^LKO^* mice or (B) *AAV-TM*-treated mice and their counterparts subjected to 90 min of hepatic ischemia followed by 24 h of reperfusion (n = 6). **P < 0.01 versus *TM^Flox^* sham or *AAV-Con* sham. Data were presented as the mean ± SD.
