# Supplementary material for: Hepatocyte-specific TMEM16A deficiency alleviates hepatic ischemia/reperfusion injury via suppressing GPX4-mediated ferroptosis
Source: Cell Death Dis. 2022 Dec 26;13(12):1072. doi: 10.1038/s41419-022-05518-w (PMC9792590; doi:10.1038/s41419-022-05518-w)

Original western blot

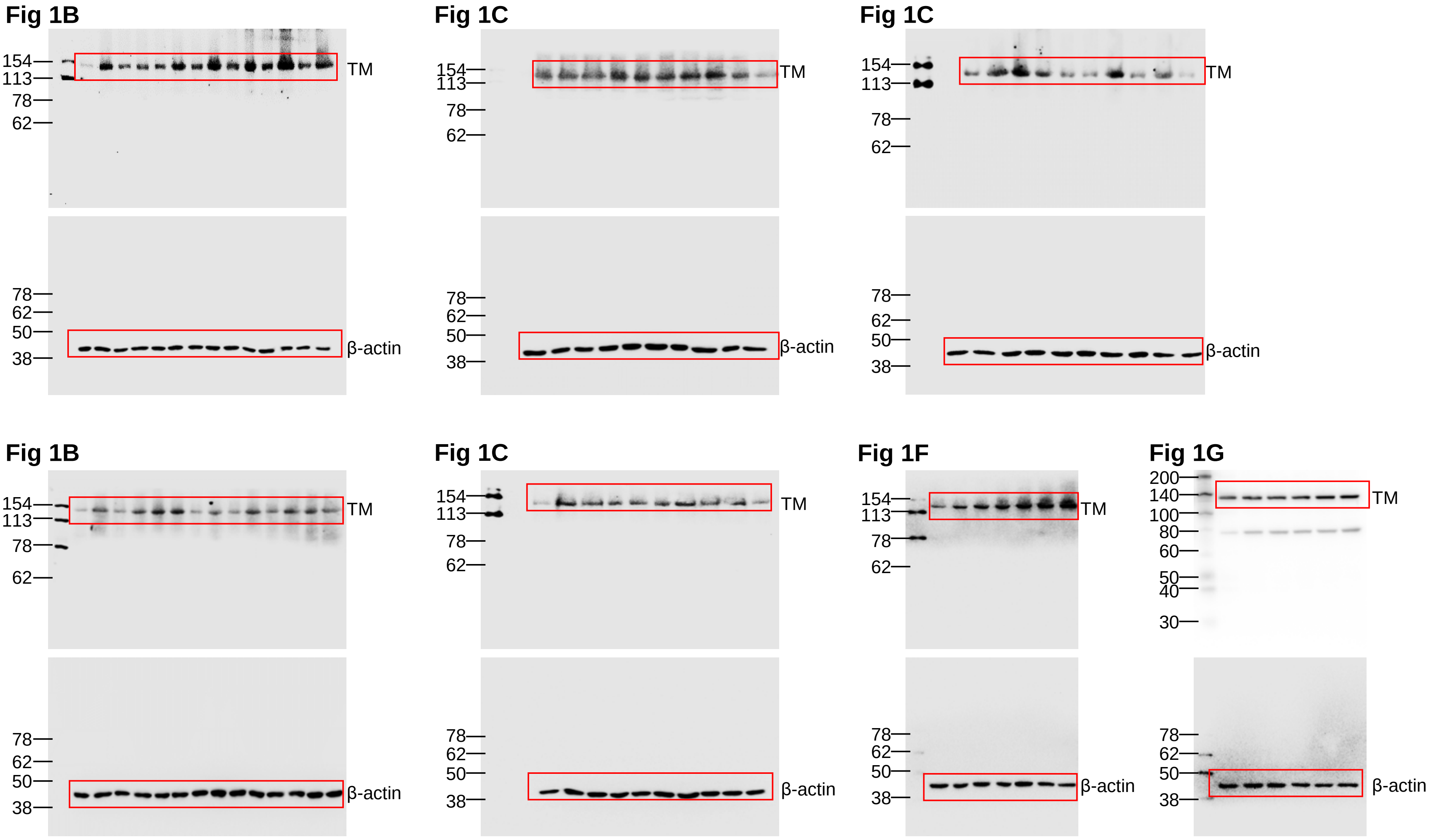

Fig 3F

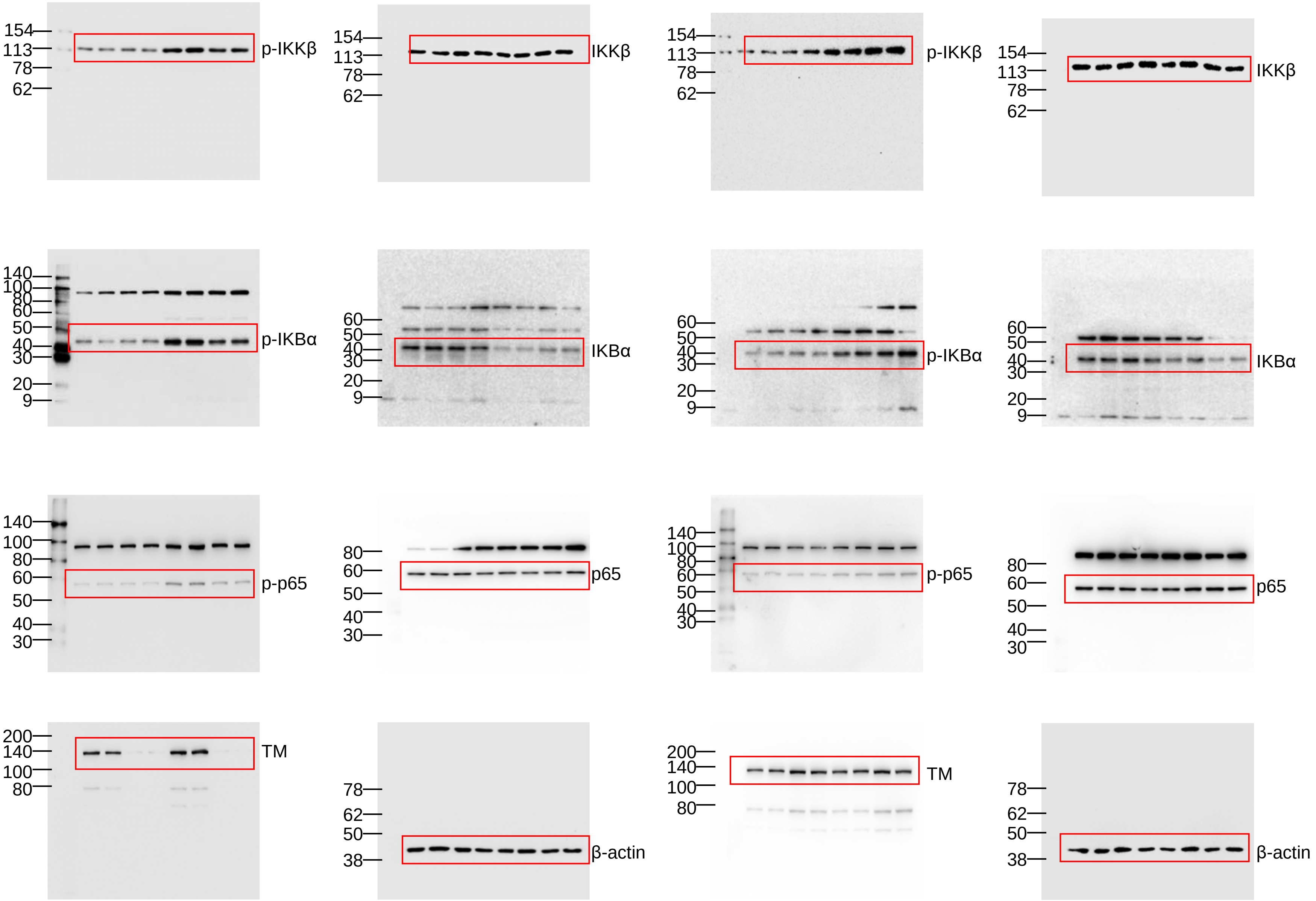

154
113
78
62
p-IKKβ
154
113
78
62
IKKβ
154
113
78
62
p-IKKβ
154
113
78
62
IKKβ
140
100
80
60
50
40
30
20
9
p-IKBα
60
50
40
30
20
9
IKBα
60
50
40
30
20
9
p-IKBα
60
50
40
30
20
9
IKBα
140
100
80
60
50
40
30
p-p65
80
60
50
40
30
p65
140
100
80
60
50
40
30
p-p65
80
60
50
40
30
p65
200
140
100
80
TM
78
62
50
38
β-actin
200
140
100
80
TM
78
62
50
38
β-actin

Fig 4D

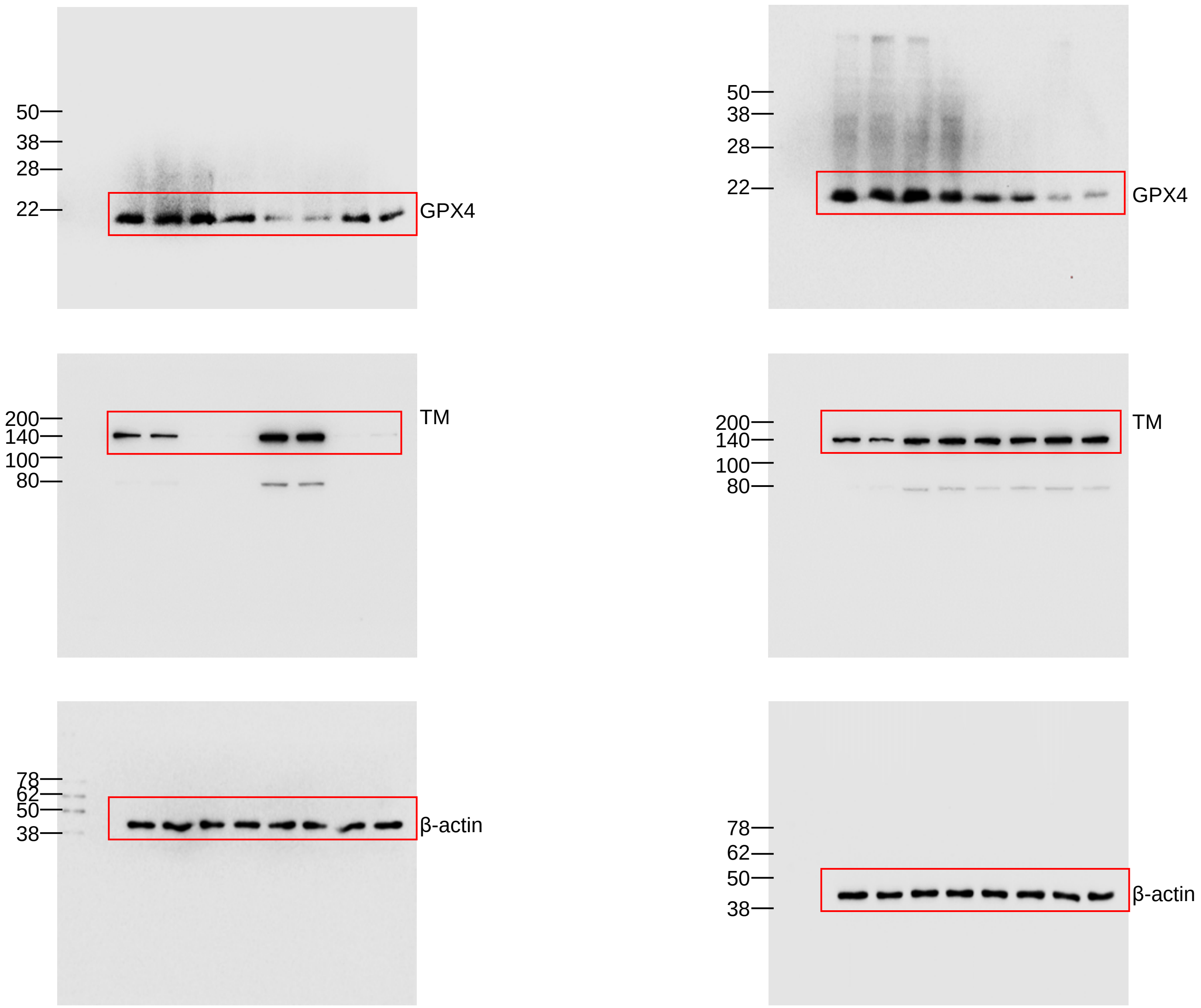
50
38
28
22
GPX4
200
140
100
80
TM
78
62
50
38
β-actin
50
38
28
22
GPX4
200
140
100
80
TM
78
62
50
38
β-actin

Fig 5H

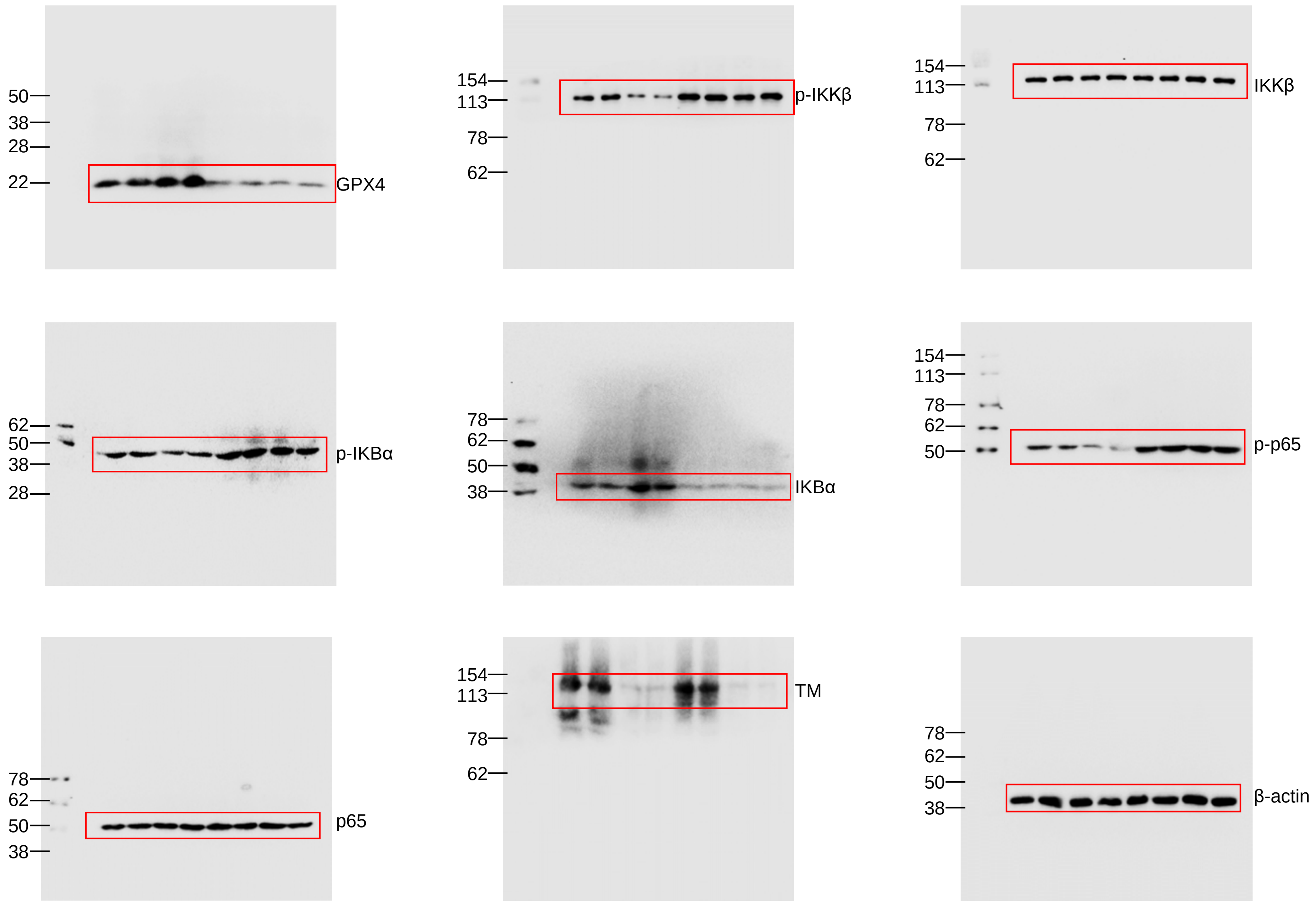
50
38
28
22
GPX4
154
113
78
62
p-IKKβ
154
113
78
62
IKKβ
62
50
38
28
p-IKBα
78
62
50
38
IKBα
154
113
78
62
50
p-p65
78
62
50
38
p65
154
113
78
62
TM
78
62
50
38
β-actin

Fig 6A

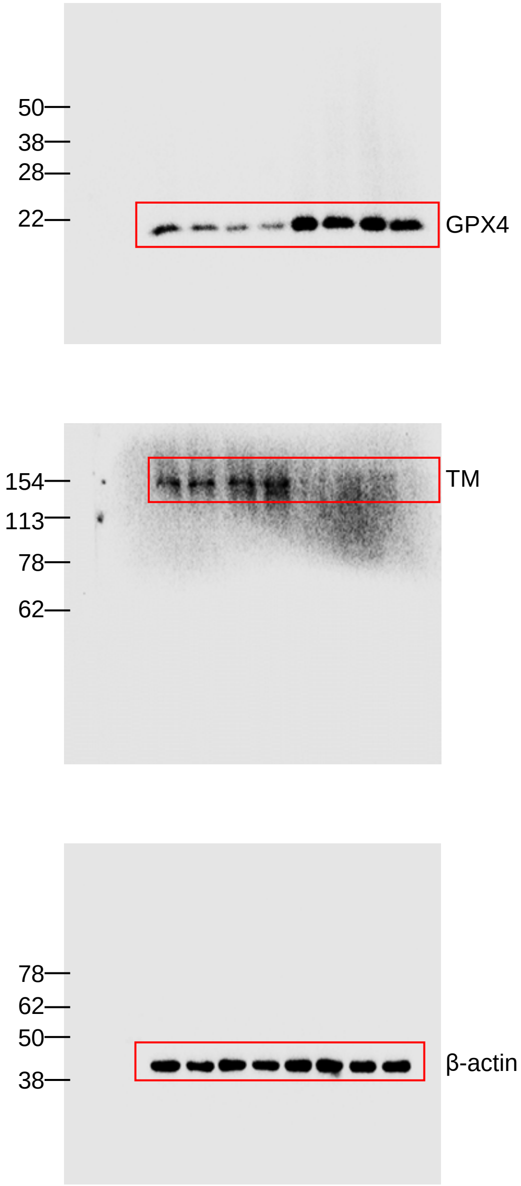

Fig 6A

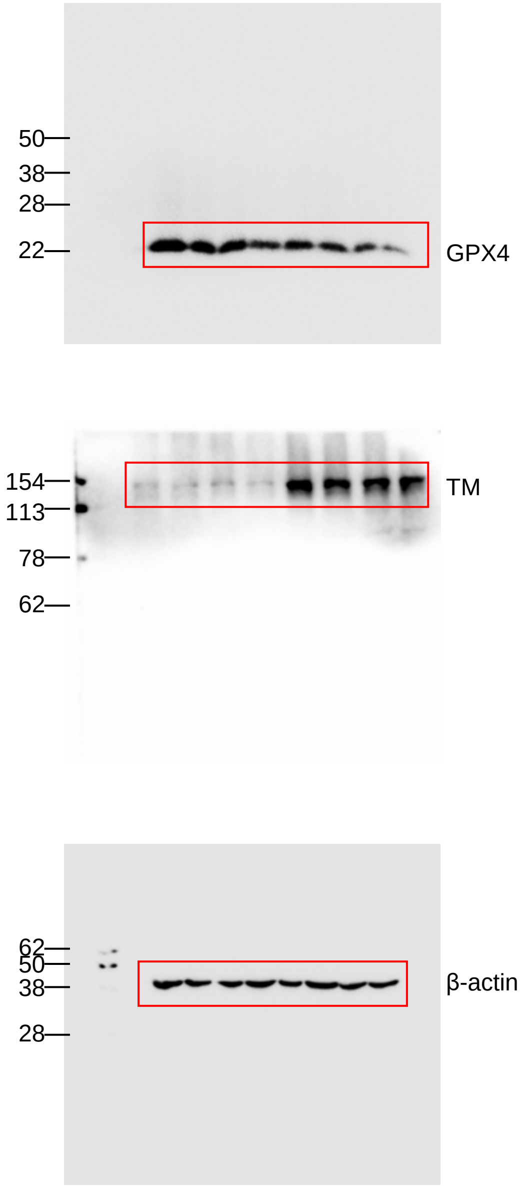

Fig 6B

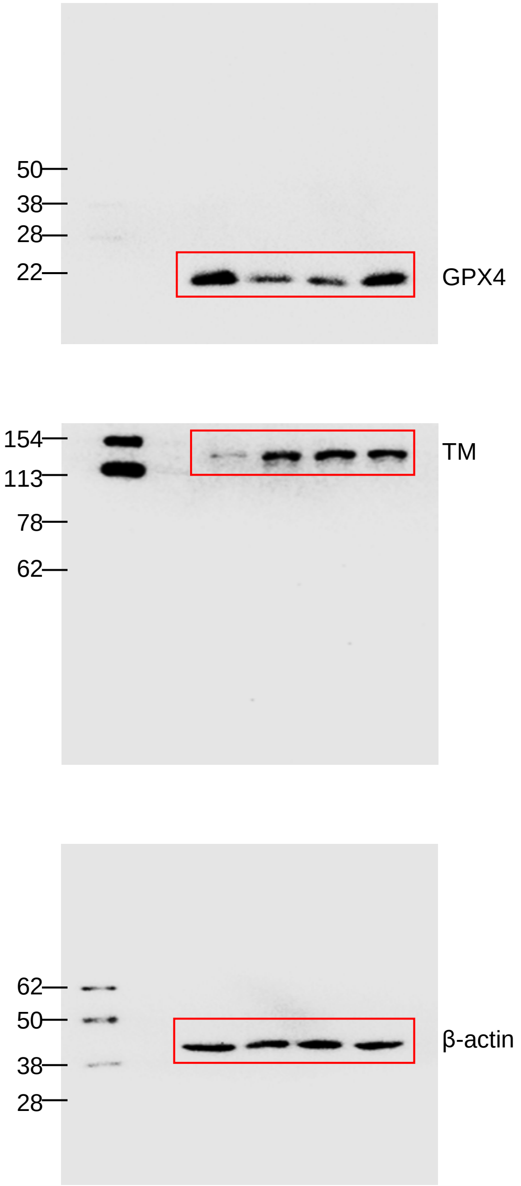

Fig 6C

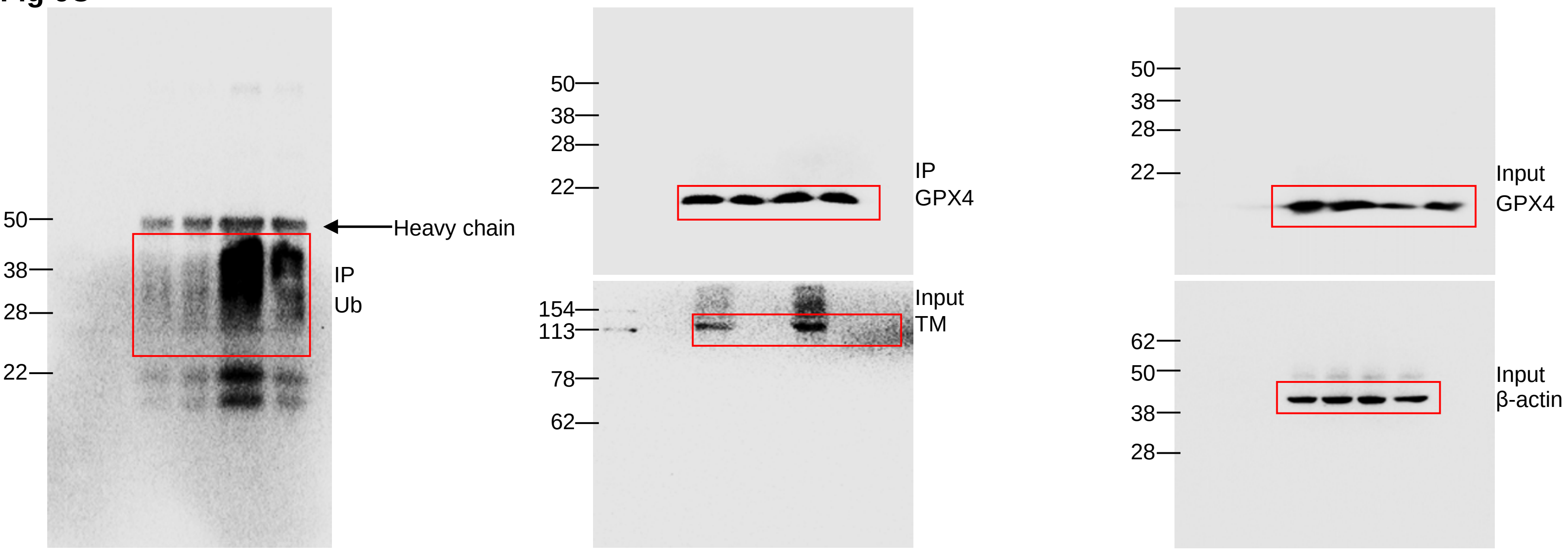
50—
38—
28—
22—
Heavy chain
IP
Ub
50—
38—
28—
22—
IP
GPX4
154—
113—
78—
62—
Input
TM
50—
38—
28—
22—
Input
GPX4
62—
50—
38—
28—
Input
β-actin

Fig 6D

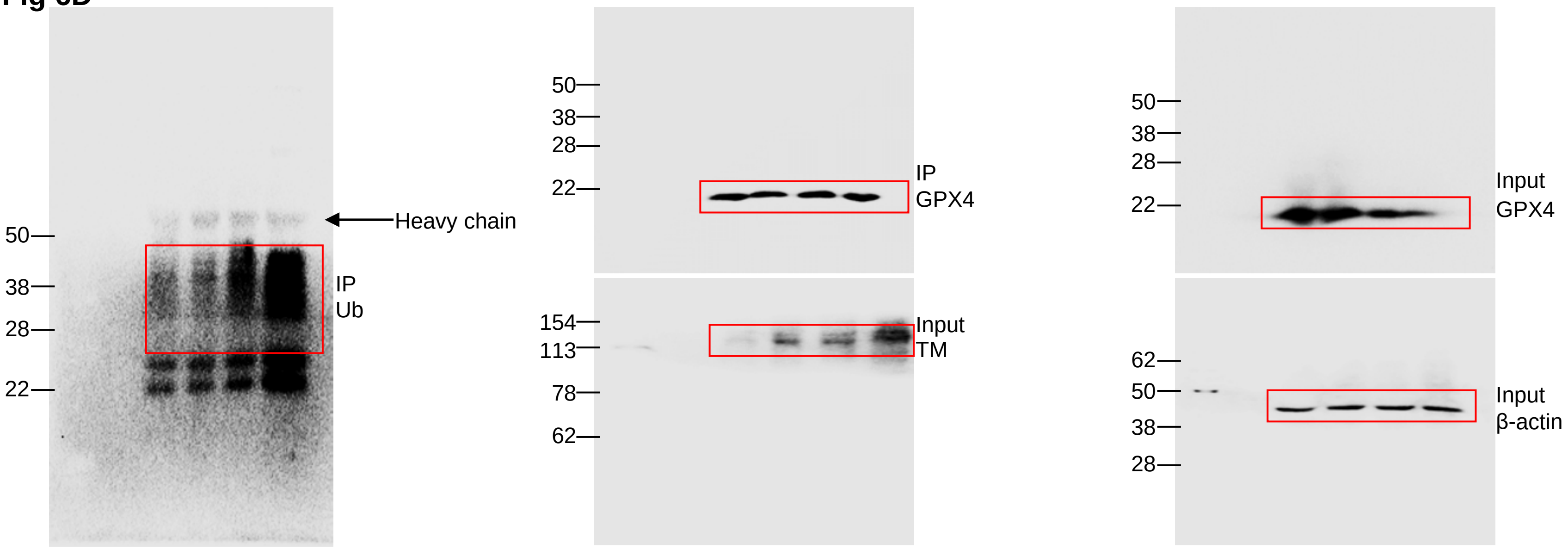
50—
38—
28—
22—
Heavy chain
IP
Ub
50—
38—
28—
22—
IP
GPX4
154—
113—
78—
62—
Input
TM
50—
38—
28—
22—
Input
GPX4
62—
50—
38—
28—
Input
β-actin

Fig 6E

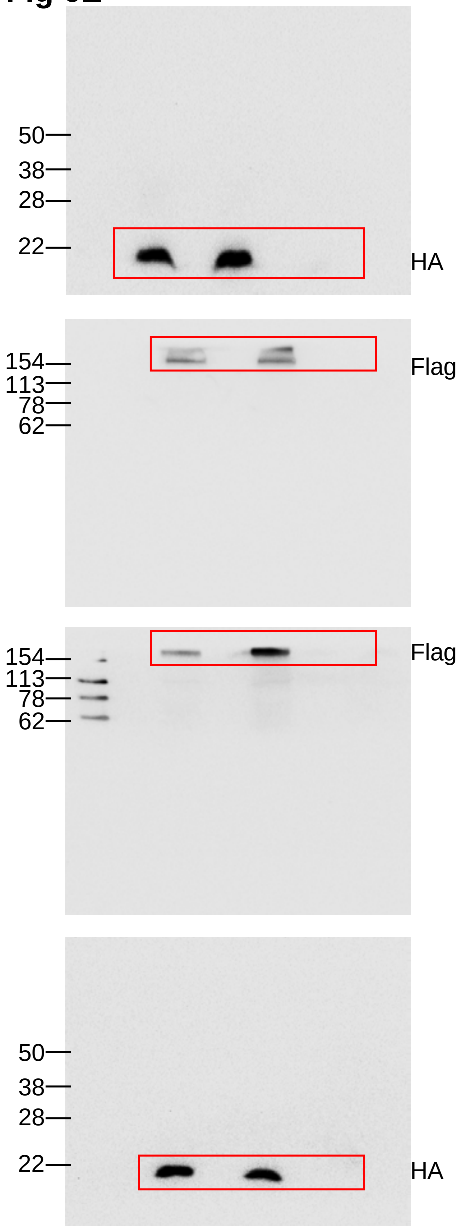
50
38
28
22
HA
154
113
78
62
Flag
154
113
78
62
Flag
50
38
28
22
HA

Fig 6F

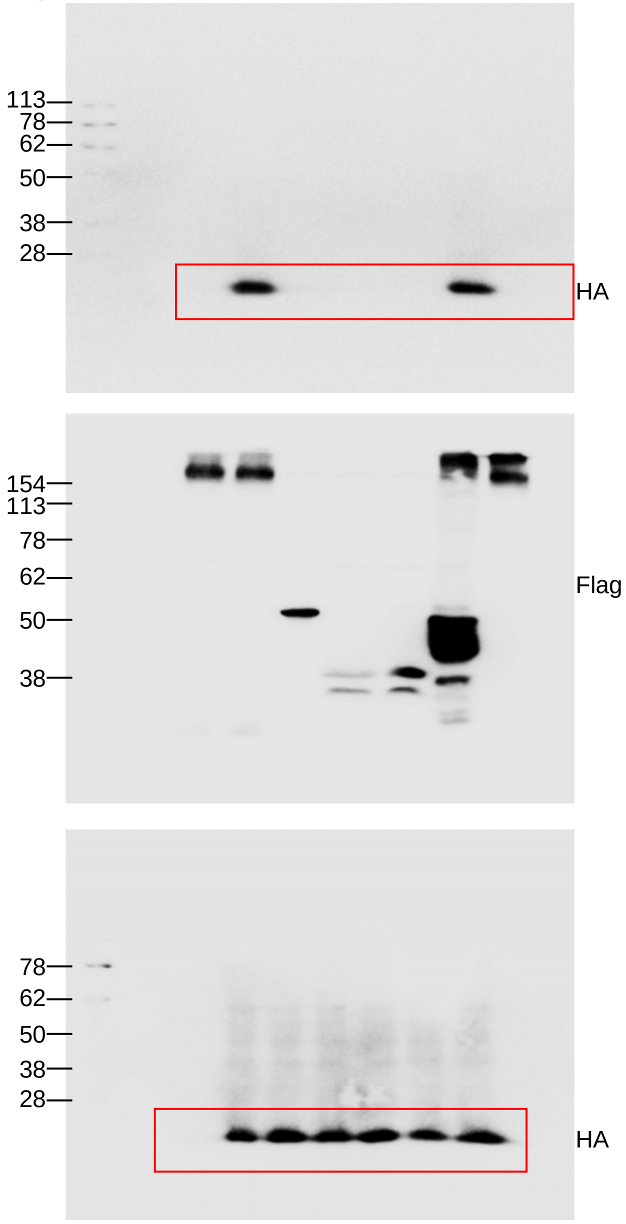
113
78
62
50
38
28
HA
154
113
78
62
50
38
Flag
78
62
50
38
28
HA

Fig 6G

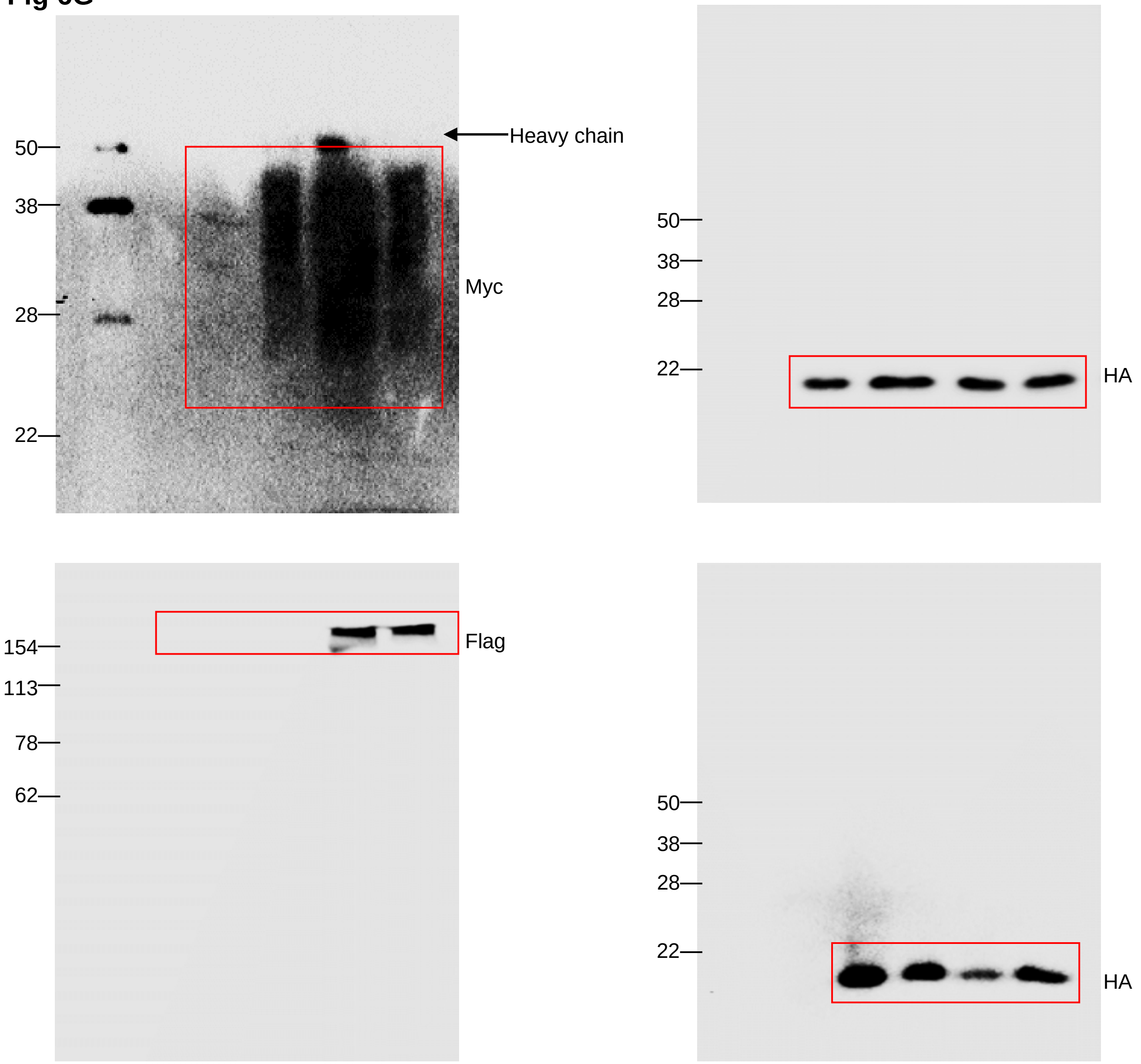

50
38
28
22
Heavy chain
Myc
50
38
28
22
HA
154
113
78
62
Flag
50
38
28
22
HA

Fig 7G

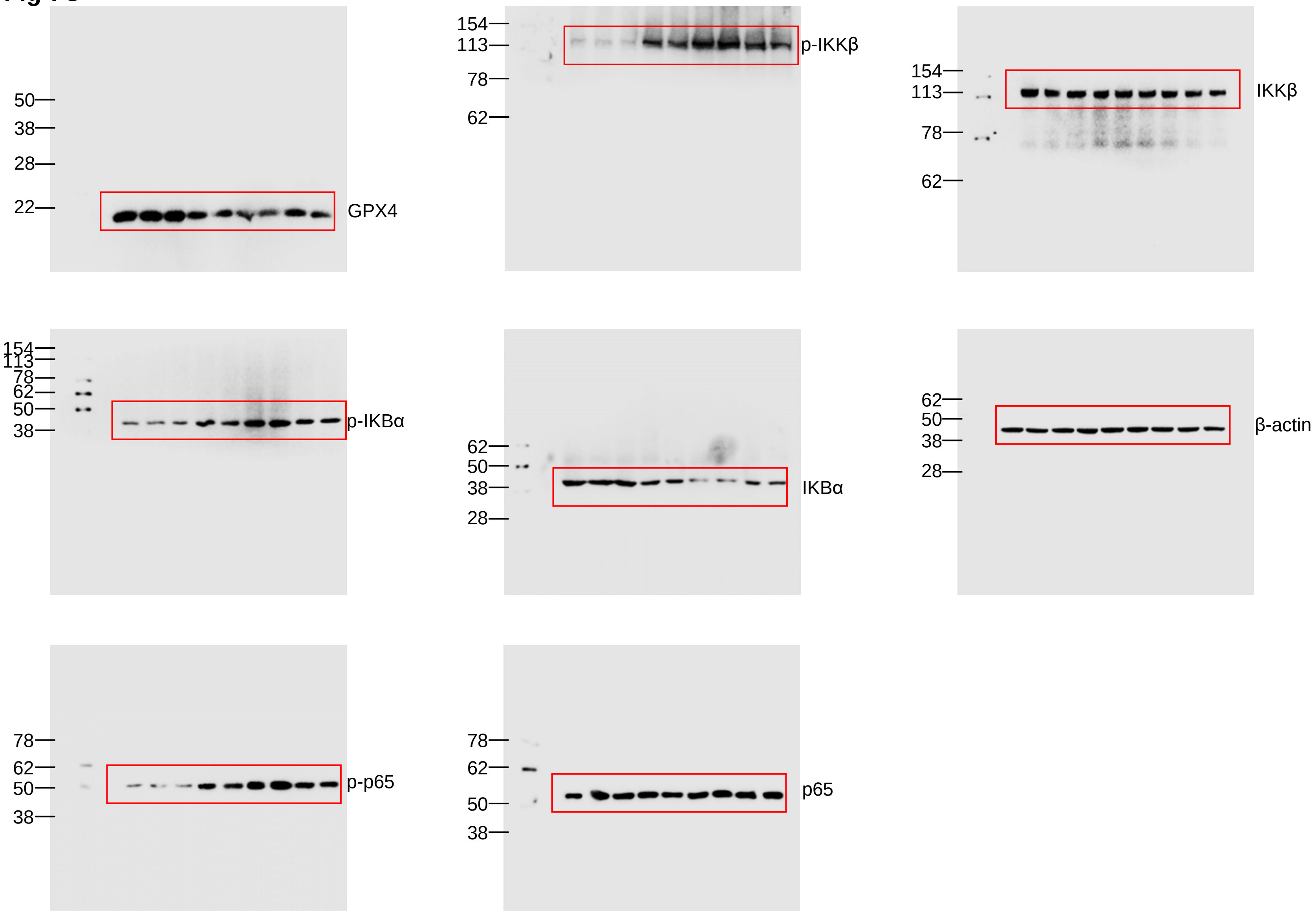
50
38
28
22
GPX4
154
113
78
62
p-IKKβ
154
113
78
62
IKKβ
154
113
78
62
50
38
p-IKBα
62
50
38
28
IKBα
62
50
38
28
β-actin
78
62
50
38
p-p65
78
62
50
38
p65

Fig S2B

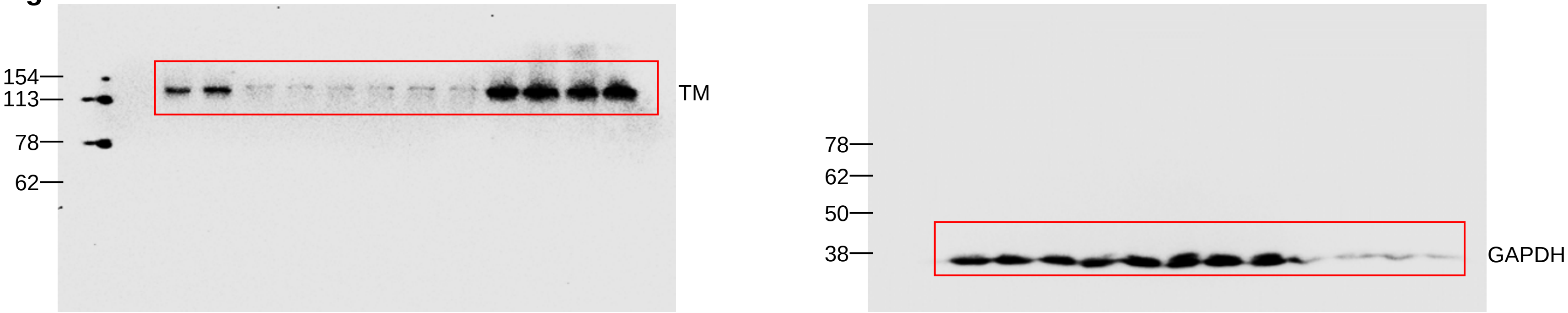

Fig S2C

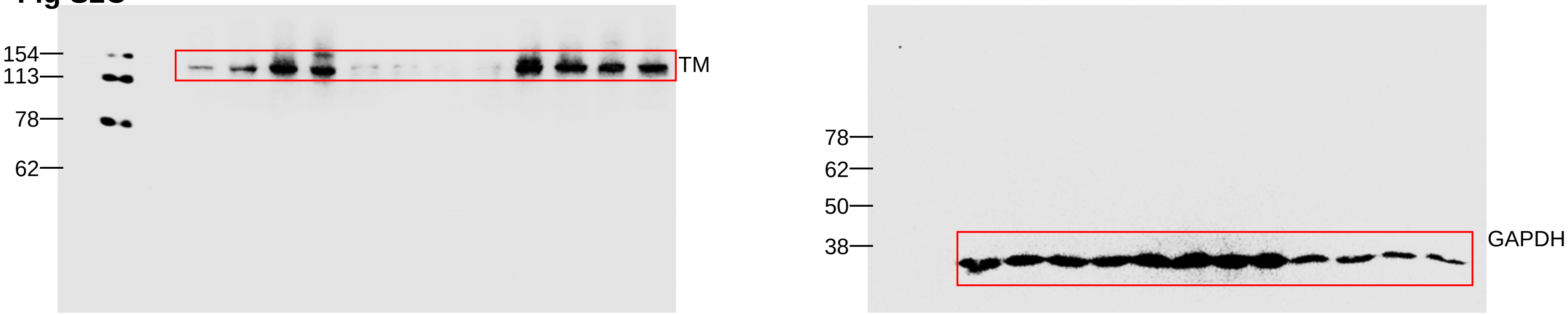

Fig S4

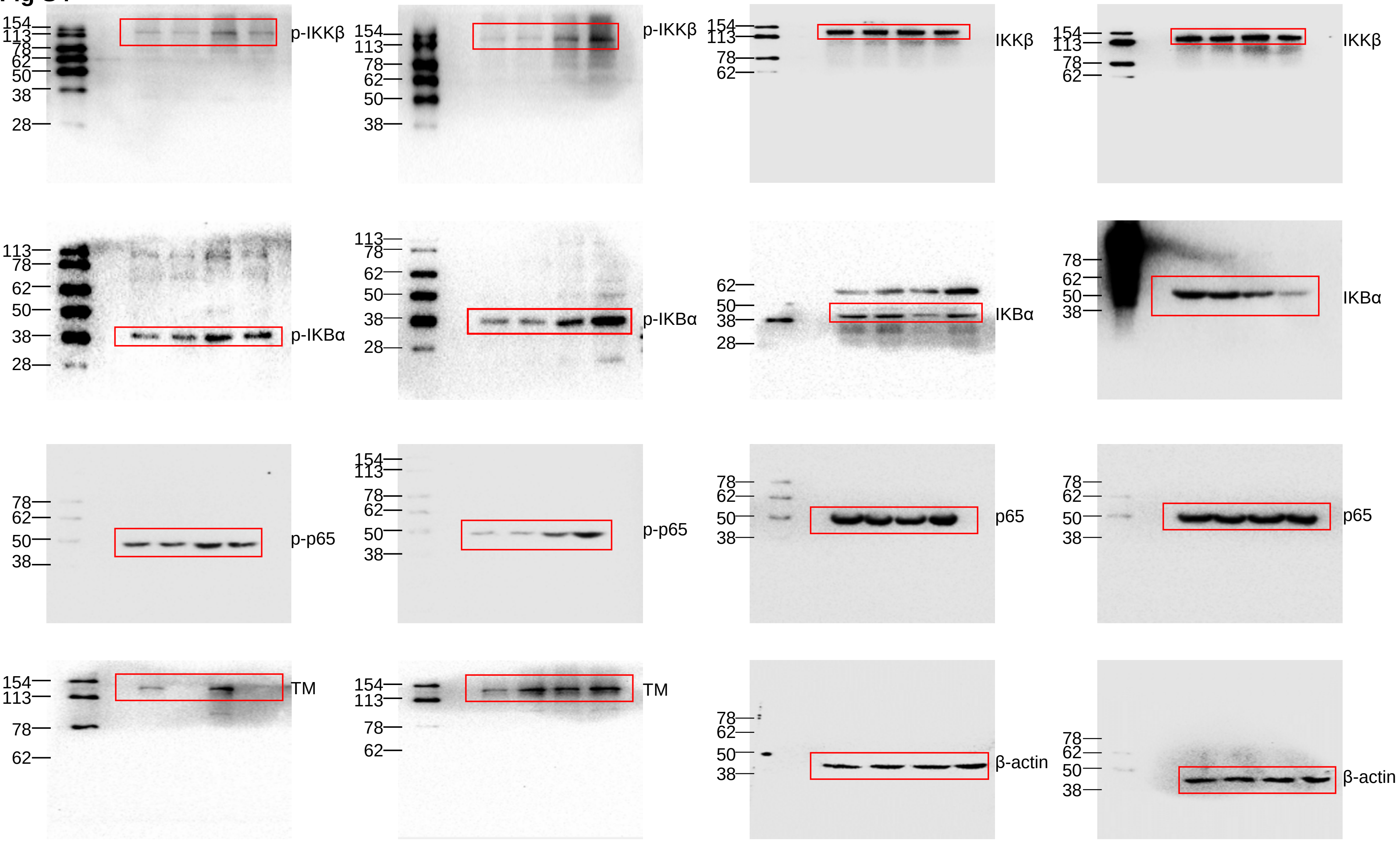
p-IKKβ
p-IKKβ
IKKβ
IKKβ
p-IKBα
p-IKBα
IKBα
IKBα
p-p65
p-p65
p65
p65
TM
TM
β-actin
β-actin
154
113
78
62
50
38
28

Fig S6

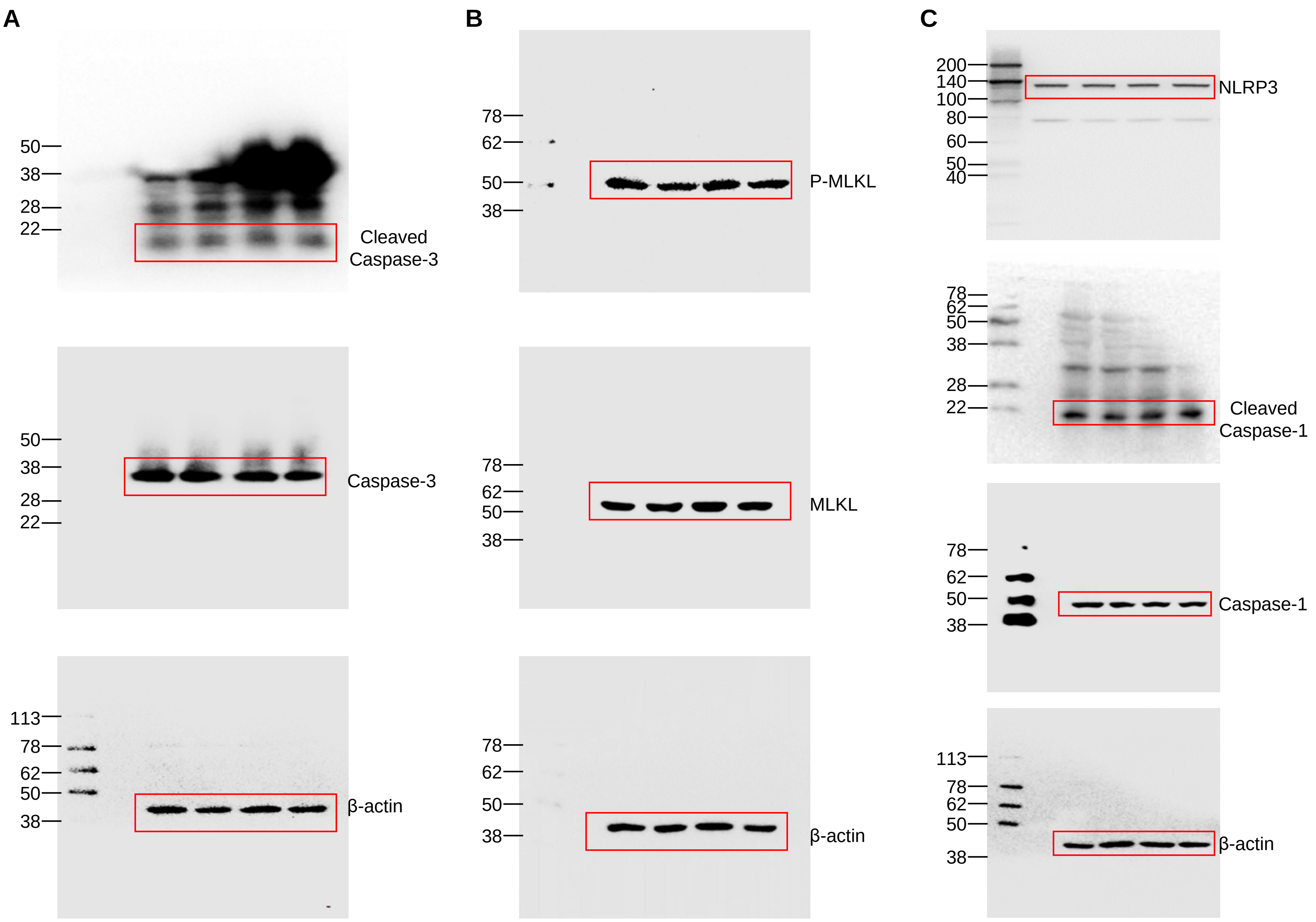
A
50
38
28
22
Cleaved Caspase-3
50
38
28
22
Caspase-3
113
78
62
50
38
β-actin
B
78
62
50
38
P-MLKL
78
62
50
38
MLKL
78
62
50
38
β-actin
C
200
140
100
80
60
50
40
NLRP3
78
62
50
38
28
22
Cleaved Caspase-1
78
62
50
38
Caspase-1
113
78
62
50
38
β-actin

Fig S7

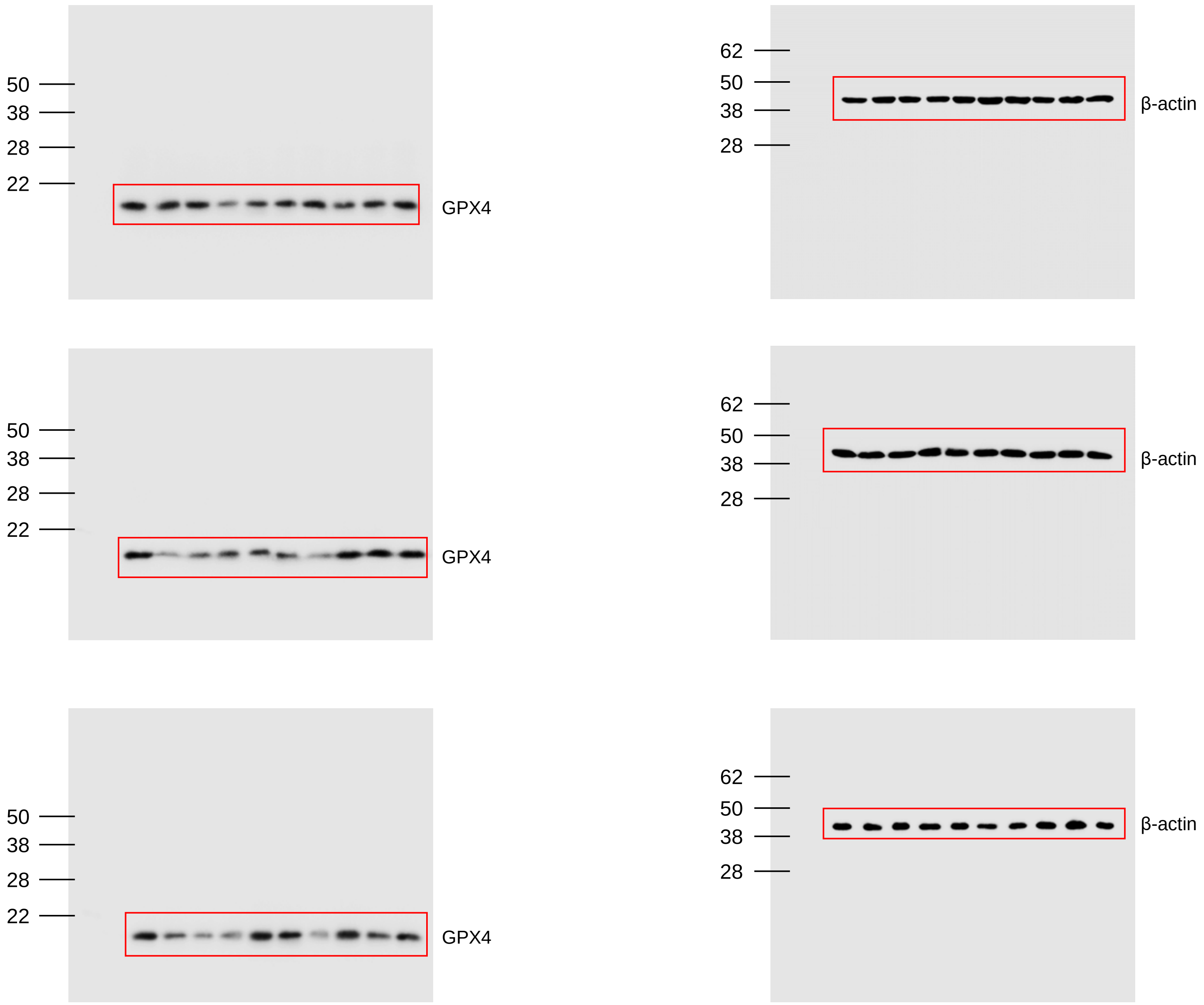
50
38
28
22
GPX4
62
50
38
28
β-actin
50
38
28
22
GPX4
62
50
38
28
β-actin
50
38
28
22
GPX4
62
50
38
28
β-actin

Fig S10

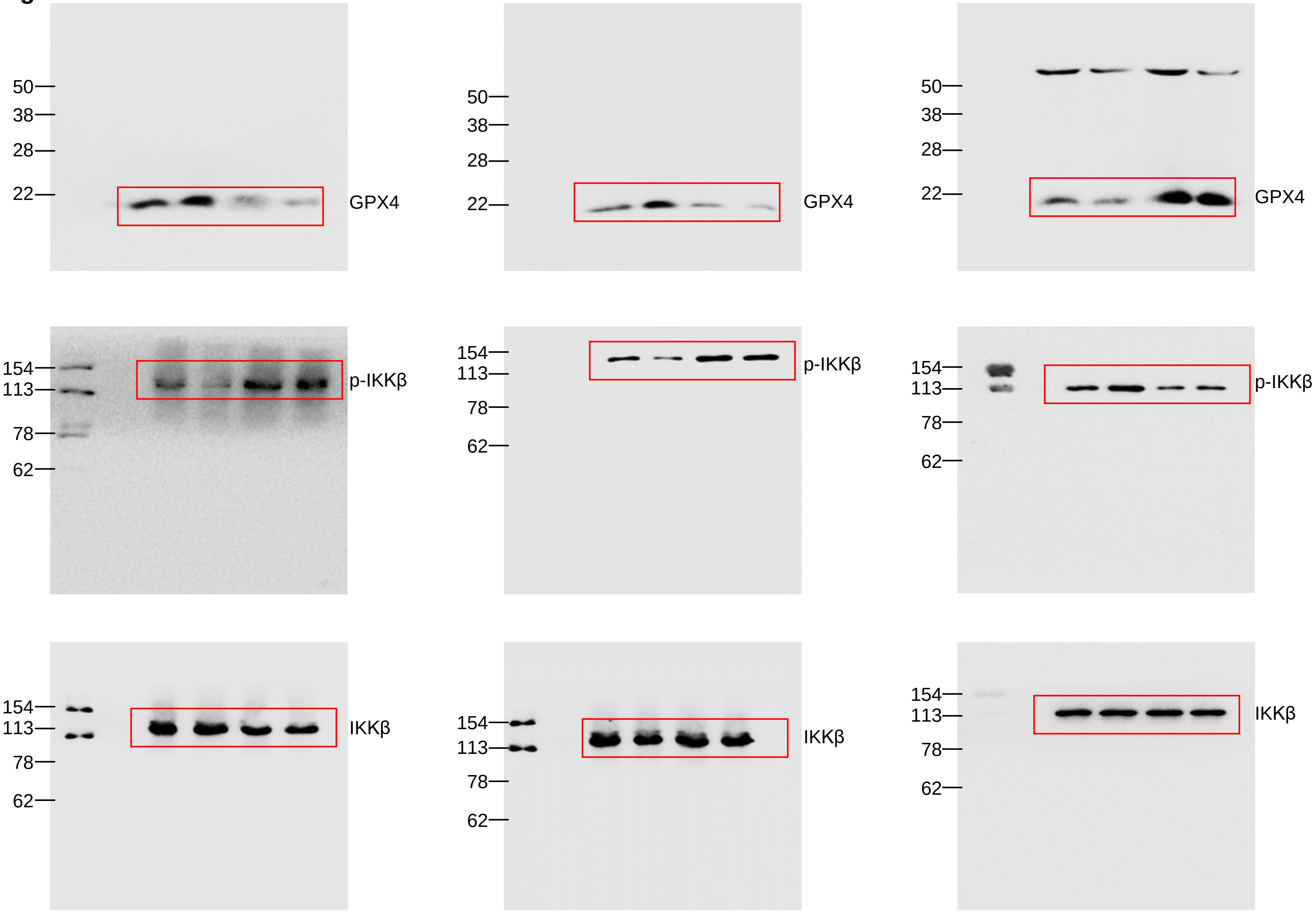
50
38
28
22
GPX4
50
38
28
22
GPX4
50
38
28
22
GPX4
154
113
78
62
p-IKKβ
154
113
78
62
p-IKKβ
154
113
78
62
p-IKKβ
154
113
78
62
IKKβ
154
113
78
62
IKKβ
154
113
78
62
IKKβ

Fig S10

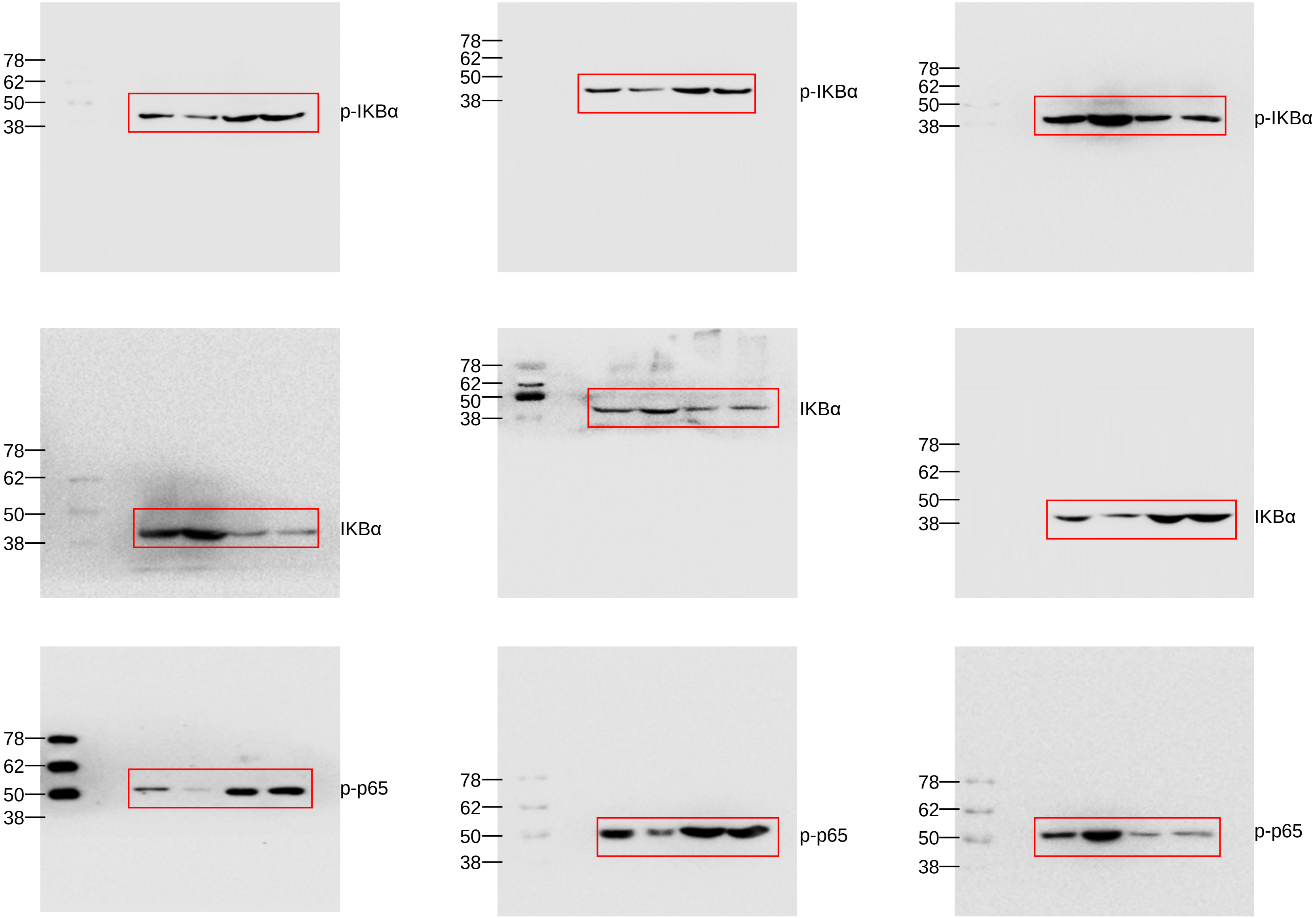
78
62
50
38
p-IKBα
78
62
50
38
p-IKBα
78
62
50
38
p-IKBα
78
62
50
38
IKBα
78
62
50
38
IKBα
78
62
50
38
IKBα
78
62
50
38
p-p65
78
62
50
38
p-p65
78
62
50
38
p-p65

Fig S10

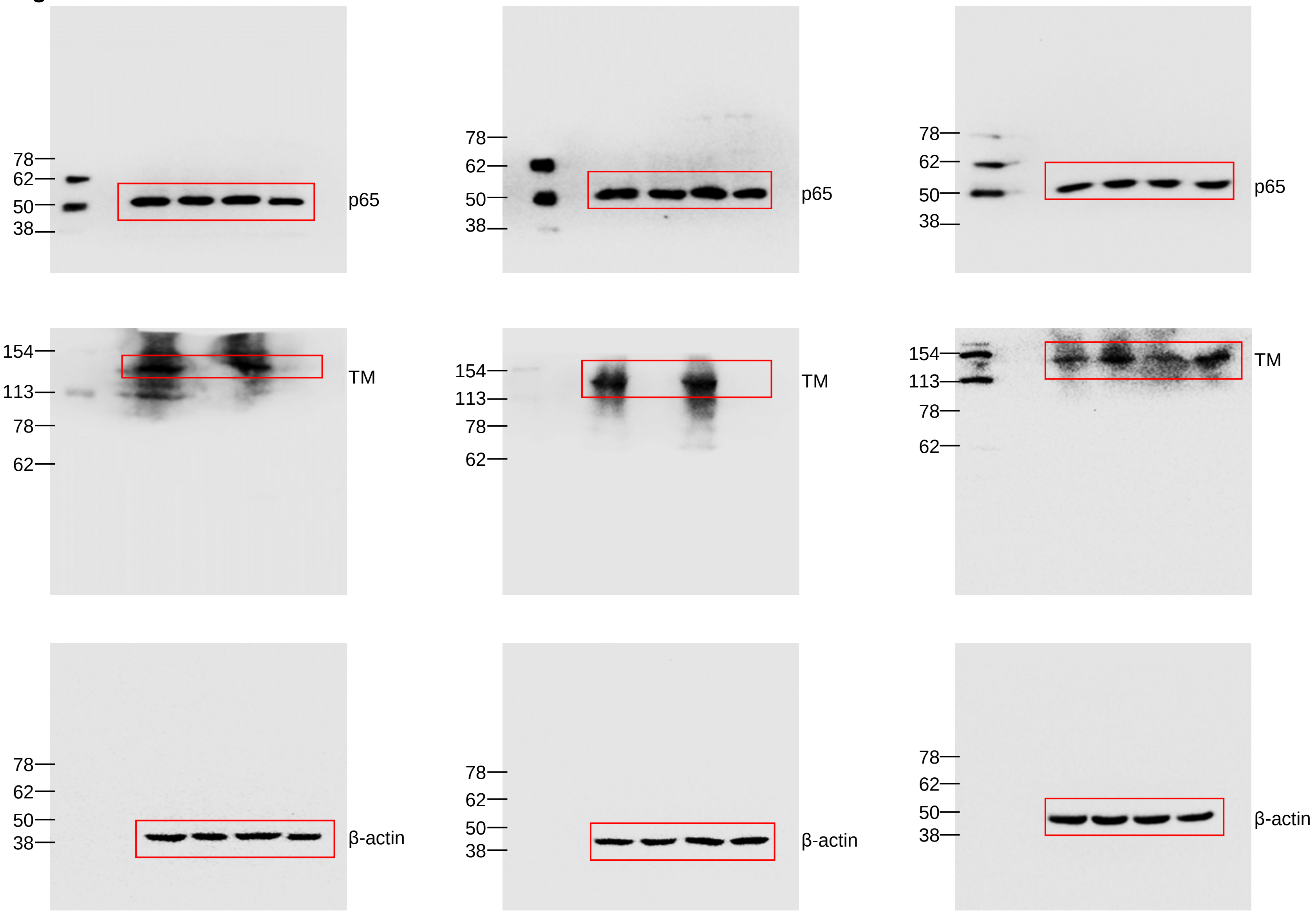
78
62
50
38
p65
78
62
50
38
p65
78
62
50
38
p65
154
113
78
62
TM
154
113
78
62
TM
154
113
78
62
TM
78
62
50
38
β-actin
78
62
50
38
β-actin
78
62
50
38
β-actin

Fig S11

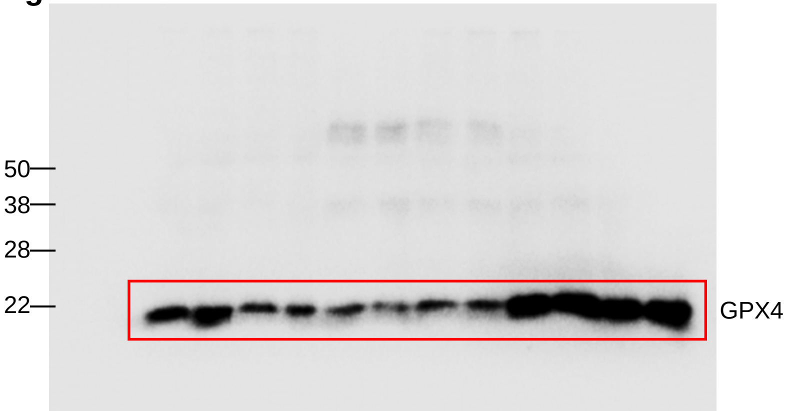

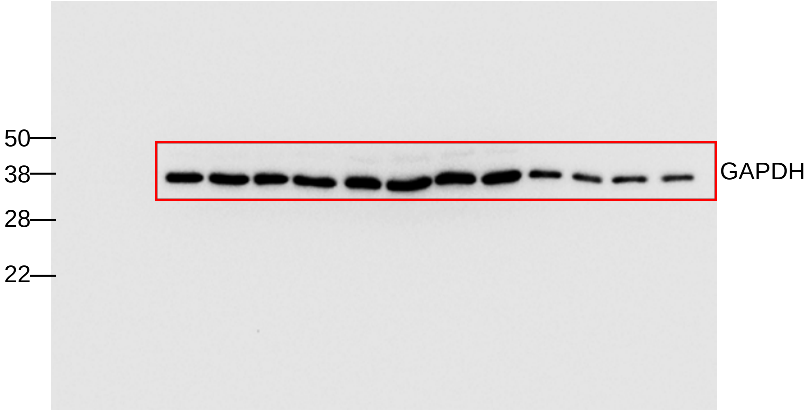

Supplement: Supplementary file 2 — Original western blots [file 41419_2022_5518_MOESM2_ESM.pdf]
